# Human-centric digital twins in industry 5.0: technologies, applications, and future directions

**DOI:** 10.3389/frobt.2026.1983733

**Published:** 2026-10-01

**Authors:** Sagheer Khan, Saifullah Tumrani, Nguyen Van Nam, Moataz Ahmed, Ashfaq Khan, Mushtaq Khan

**Affiliations:** 1 SDAIA-KFUPM Joint Research Center for Artificial Intelligence, KFUPM, Dhahran, Saudi Arabia; 2 Information and Computer Science Department, King Fahd University of Petroleum & Minerals (KFUPM), Dhahran, Saudi Arabia; 3 School of Engineering and Built Environment, Sheffield Hallam University, Sheffield, United Kingdom; 4 Additive Manufacturing Research and Innovation Center (AMRIC), Mechanical Engineering Department, College of Engineering, Prince Mohammad Bin Fahd University, Al Khobar, Saudi Arabia

**Keywords:** adaptive robotics, human intention prediction, human sensing, human-centric digital twin, human-robot collaboration, industry 5.0

## Abstract

Industry 5.0 (I5.0) emphasizes industrial technologies that are human-centric, sustainable, and resilient. Human-Centric Digital Twins (HCDTs) support this transition by integrating worker data with robots, machines, tasks, and industrial environments. However, the relevant research remains fragmented across human modelling, Human–Robot Collaboration, Artificial Intelligence (AI), simulation, and Digital Twin (DT) research. Moreover, systems that use human data or adaptive automation are often described as human-centric without demonstrating measurable benefits to workers. This review addresses these limitations by distinguishing HCDTs from conventional human models, Human DTs, and collaborative robotic systems. It critically examines human-focused sensing, multi-modal data integration, biomechanical modelling, intention and motion prediction, robotic simulation, optimization, reinforcement learning, immersive interfaces, industrial connectivity, and data governance. Based on this analysis, a literature-derived conceptual framework is proposed that connects sensing, integration, human modelling, analytics, simulation, decision-making, adaptation, human interaction, and governance. The framework organizes the functional requirements of HCDTs and is intended to inform future system design and comparison; its practical applicability and effectiveness require further validation. The review argues that HCDTs must extend beyond worker monitoring and productivity maximization: human data should inform industrial decisions that produce measurable improvements in safety, ergonomics, wellbeing, autonomy, accessibility, or skill development. Such systems should also uphold privacy, transparency, uncertainty awareness, explainability, and appropriate human authority. By linking technological capabilities to explicit worker outcomes, this synthesis enables researchers and engineers to assess the human-centeredness of HCDT implementations and supports the development of trustworthy systems for I5.0.

## 1 Introduction

Industry 5.0 (I5.0) emerged shortly after the widespread adoption of Industry 4.0 (I4.0), creating debate over whether it constitutes a distinct industrial revolution, an extension of I4.0, or a hybrid framework in which both paradigms coexist (Golovianko et al., 2023). I4.0 is predominantly technology-driven, emphasizing digitization, automation, cyber-physical systems, artificial intelligence, the Internet of Things, cloud computing, and interconnected production. Although these technologies have substantially improved productivity, flexibility, accuracy, and real-time decision-making, I4.0 has been criticized for placing insufficient emphasis on workers, societal value, environmental sustainability, and industrial resilience (Zizic et al., 2022; Xu et al., 2021). I5.0 responds to these limitations by retaining the technological foundations of I4.0 while expanding industrial objectives beyond efficiency and profitability.

The central principle of I5.0 is that technology should support human capabilities rather than merely replace human workers. This approach combines human creativity, intuition, experience, adaptability, and problem-solving ability with the precision, consistency, speed, and analytical capability of intelligent machines (Nahavandi, 2019). The European Commission formalized this perspective through reports describing the enabling technologies and strategic foundations of I5.0 (European Commission, 2020; 2021). According to this framework, I5.0 is built on three interconnected pillars: human-centricity, sustainability, and resilience. Human-centricity places worker needs, rights, safety, and wellbeing at the center of production. Sustainability aligns industrial operations with environmental limits, resource efficiency, waste reduction, circular production, and reduced emissions. Resilience concerns the capacity of organizations, production systems, and supply chains to anticipate, withstand, adapt to, and recover from unexpected disruptions.

I5.0 therefore represents a shift from technology-centered automation towards value-driven industrial development. It seeks to establish flexible and socially responsible manufacturing environments in which human workers retain meaningful roles and appropriate control over intelligent production systems (Wolniak, 2023). This transition may be especially relevant to small and medium-sized enterprises, for which complete automation can be technically complex and financially difficult to implement. Human–machine collaboration can provide these organizations with greater production flexibility and enable them to respond more rapidly to changing customer requirements while retaining human knowledge and judgment within the production process (Kralj and Aralica, 2023).

Collaborative robots, commonly known as cobots, are an important component of this human-centric production model. Unlike conventional industrial robots that generally operate independently or within restricted areas, cobots are designed to share tasks and workspaces with human operators. Their purpose is to augment human capabilities by performing repetitive, physically demanding, hazardous, or highly precise tasks while workers contribute contextual understanding, creativity, and decision-making (Zafar et al., 2024). However, closer interaction between workers and intelligent machines introduces additional risks. Humans and automated systems may interpret the same operational situation differently, resulting in observation, interpretation, or control-action conflicts (Wen et al., 2023; Arunthavanathan et al., 2024). Consequently, human authority, communication mechanisms, system transparency, and safe intervention procedures must be clearly established.

The relationship between worker safety and environmental sustainability also requires careful consideration. Increasing the safety of human–robot collaboration may require additional sensors, protective systems, computing resources, control equipment, and energy consumption. This creates a possible trade-off between environmental performance and the safety requirements of collaborative workplaces (Bonello et al., 2024). I5.0 must therefore avoid treating human-centric, sustainability, and resilience as independent objectives. These principles must be evaluated together so that improvements in one area do not create unacceptable consequences in another.

DTs provide an important technological foundation for integrating these objectives. A DT is a continuously updated virtual representation of a physical asset, process, or production environment. It receives operational data from the physical system and uses models, simulations, and analytical methods to support production optimization, predictive maintenance, operator training, adaptive control, and future system design. Previous research has identified DT definitions, applications, integration, modelling, and data as central areas for evaluating DT development approaches (Alnowaiser and Ahmed, 2023). Within I5.0, DTs can strengthen human–machine collaboration by providing workers with contextual information and allowing alternative operating conditions to be evaluated before decisions are implemented in the physical system (Barata and Kayser, 2024).

The DT concept can also be extended from machines and processes to humans. A HDT is intended to represent relevant physiological, cognitive, behavioral, and emotional characteristics of a worker. Early research has examined whether such a representation is technically achievable and how it could mediate interactions between human operators and intelligent industrial systems (Wang, 2021). More recent studies describe HDTs as an enabling technology for human-centric manufacturing because they can incorporate worker capabilities, limitations, fatigue, workload, and wellbeing into the design and operation of industrial systems (Wang B. et al., 2024). Nevertheless, the collection and processing of human data introduce ethical concerns regarding informed consent, privacy, surveillance, algorithmic bias, confidentiality, and ownership of personal information.

I5.0 should consequently be understood as a socio-technical industrial framework rather than simply another stage of automation. Its success depends on the coordinated development of technology, human skills, organizational structures, ethical governance, occupational safety, environmental responsibility, and system resilience. Although many of its enabling technologies originated in I4.0, I5.0 changes the purpose for which these technologies are applied. The objective is no longer limited to maximizing production efficiency; it is to create industrial systems that are productive, human-centered, sustainable, resilient, and capable of generating broader value for workers, organizations, and society.

This review goes beyond classifying enabling technologies by defining the distinguishing characteristics of a Human-Centric Digital Twin and proposing a literature-derived conceptual framework for closed-loop integration. The framework connects multi-modal human sensing, AI-based prediction, simulation, industrial systems, adaptive robotics, human oversight, and intended worker-centered outcomes. It synthesizes architectural requirements identified in the reviewed literature rather than reporting an empirically validated implementation. Additionally, human-centricity refers to the overarching I5.0 principle, human-centered design refers to the established participatory design approach, and worker-centered describes outcomes, benefits, and evaluation criteria directly related to workers.

The review is organized around four research questions:

RQ1: What distinguishes Human-Centric Digital Twins from related human models, Digital Twins, and HRC systems in I5.0?

RQ2: How can multi-modal sensing, AI, industrial data, and simulation be integrated into an I5.0 HCDT architecture?

RQ3: How can HCDTs enable trustworthy, closed-loop adaptation of robots, tasks, and manufacturing operations in I5.0?

RQ4: What requirements ensure that I5.0 HCDTs deliver measurable improvements in worker safety, ergonomics, wellbeing, autonomy, and skills?

## 2 Literature selection and title-term mapping

Relevant publications were identified through searches of Scopus, Web of Science Core Collection, IEEE Xplore, ScienceDirect, SpringerLink, and Google Scholar, supplemented by backward citation searching. Records were screened for relevance to Human-Centric Digital Twins, Industry 5.0, human modelling, human-robot collaboration, enabling technologies, and worker-centered outcomes, resulting in 134 included publications. Title-term analysis was then conducted in Python 3.12.13 using pandas 2.2.3 and NetworkX 3.6.1. Titles were converted to lowercase and cleaned by removing LaTeX commands, punctuation, and repeated whitespace and by standardizing hyphens. Singular/plural forms and equivalent expressions were merged using a predefined synonym dictionary, including “digital twins”/“digital twin”, “human-cobot collaboration”/“human-robot collaboration”, and “cobot”/“collaborative robot”. Each standardized term was counted once per title, with a minimum-occurrence threshold of one publication. Two terms were linked when they appeared in the same title, and total link strength was calculated as the sum of all title-level co-occurrences involving each term. The thematic groups were assigned conceptually rather than through automated clustering; therefore, clustering and resolution parameters were not applicable.

In the resulting network maps, each circle represents a standardized term identified in the publication titles. The size of each circle corresponds to the frequency with which the term appeared, while the thickness of the line connecting two circles represents their co-occurrence frequency. Terms associated with the core concepts of HCDTs were organized by colour into thematic groups covering human-robot systems, AI and modelling, human state and safety, interfaces and infrastructure, and trust and governance.

The analysis identified Digital Twin, Industry 5.0, Human-Robot Collaboration, I4.0, and Artificial Intelligence as prominent title terms and revealed their relationships with simulation, sustainability, human-centricity, Human Digital Twins, cybersecurity, Cyber-Physical Systems, virtual and augmented reality, and smart manufacturing. Table 1 reports the title-term occurrence frequencies and total link strengths, together with the distributions of the selected publications by publication type and publication period. Figure 1 presents the complete title-term co-occurrence network, while Figure 2 emphasizes the relationships among Digital Twins, Human-Robot Collaboration, Industry 5.0, and associated human-centric industrial concepts by fading secondary terms and connections.

**TABLE 1:** Quantitative characteristics and title-term mapping results for the selected publications.

Panel A: title-term mapping results					
Term	Occurrences	Link strength	Term	Occurrences	Link strength
Digital twin	51	55	Virtual reality	5	5
Industry 5.0	25	29	Cyber-physical systems	4	5
Human-robot collaboration	14	17	Artificial intelligence	4	4
Industry 4.0	11	14	Machine learning	4	4
Human-centricity	8	12	Cybersecurity	4	4
Sustainability	6	13	Reinforcement learning	4	4
Human digital twin	6	13	Mixed reality	3	5
Simulation	6	9	Smart manufacturing	3	6
Human–Robot interaction	6	4	Blockchain	3	4
Deep learning	5	5	Collaborative robot	3	4
Resilience	4	9	Worker safety	2	5
Augmented reality	4	5	Brain–Computer interface	2	2
Wellbeing	1	4	Motion prediction	1	2
Human modelling	1	3	Human factors	1	3
Ergonomics	1	1			

Panel B: publication-type distribution					
Publication type	Number	Percentage	Publication type	Number	Percentage
Journal article	121	90.3%	Conference paper	6	4.5%
Technical report	2	1.5%	Doctoral thesis	2	1.5%
Other professional publication	2	1.5%	Book chapter	1	0.7%

Panel C: publication-period distribution					
Publication period	Number	Percentage	Publication period	Number	Percentage
Before 2015	11	8.2%	2015–2019	23	17.2%
2020–2022	54	40.3%	2023–2024	30	22.4%
2025–2026	16	11.9%	Total	134	100.0%

**FIGURE 1:**
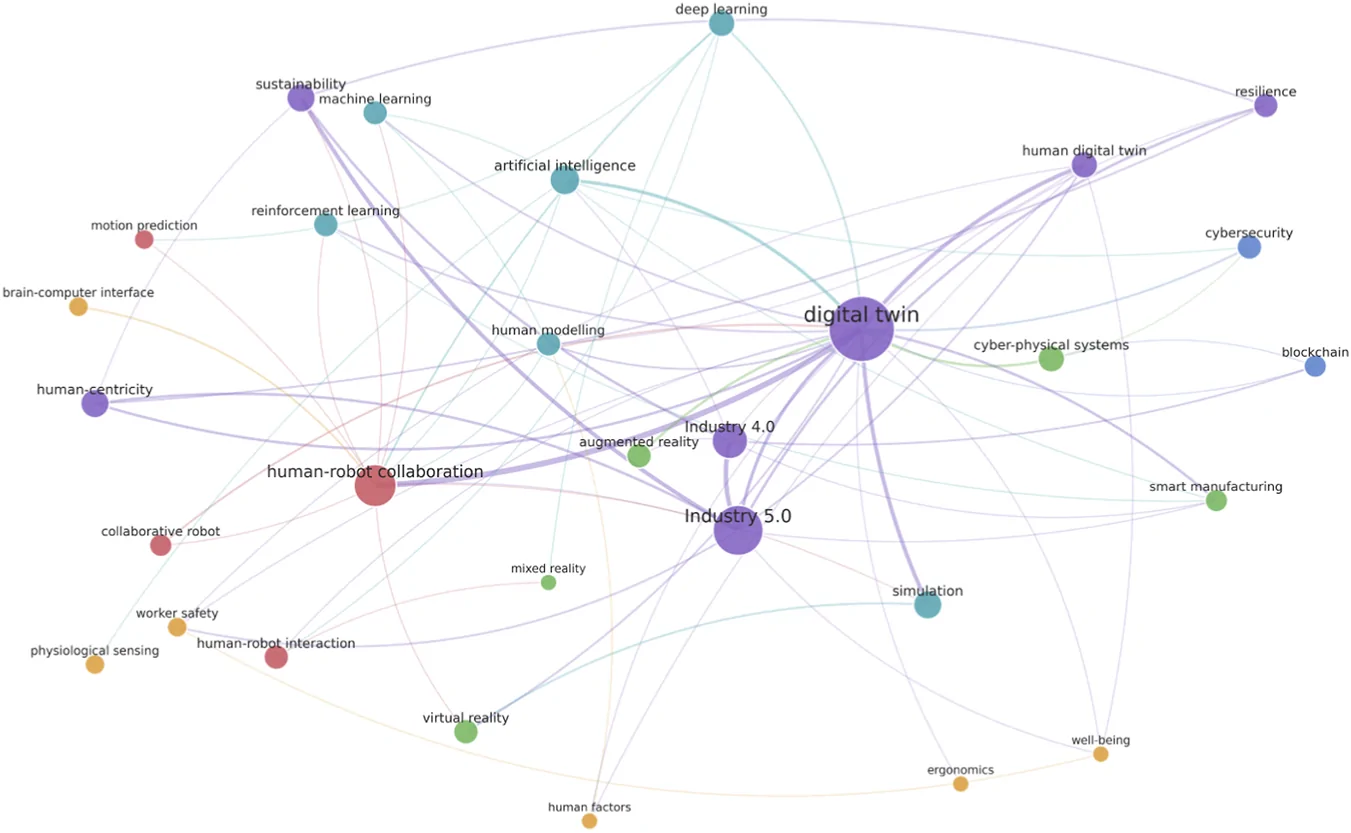
Complete title-term co-occurrence network of the 134 publications selected for this review. Node size represents occurrence frequency, line thickness indicates co-occurrence strength, and colours distinguish the principal HCDT thematic groups.

**FIGURE 2:**
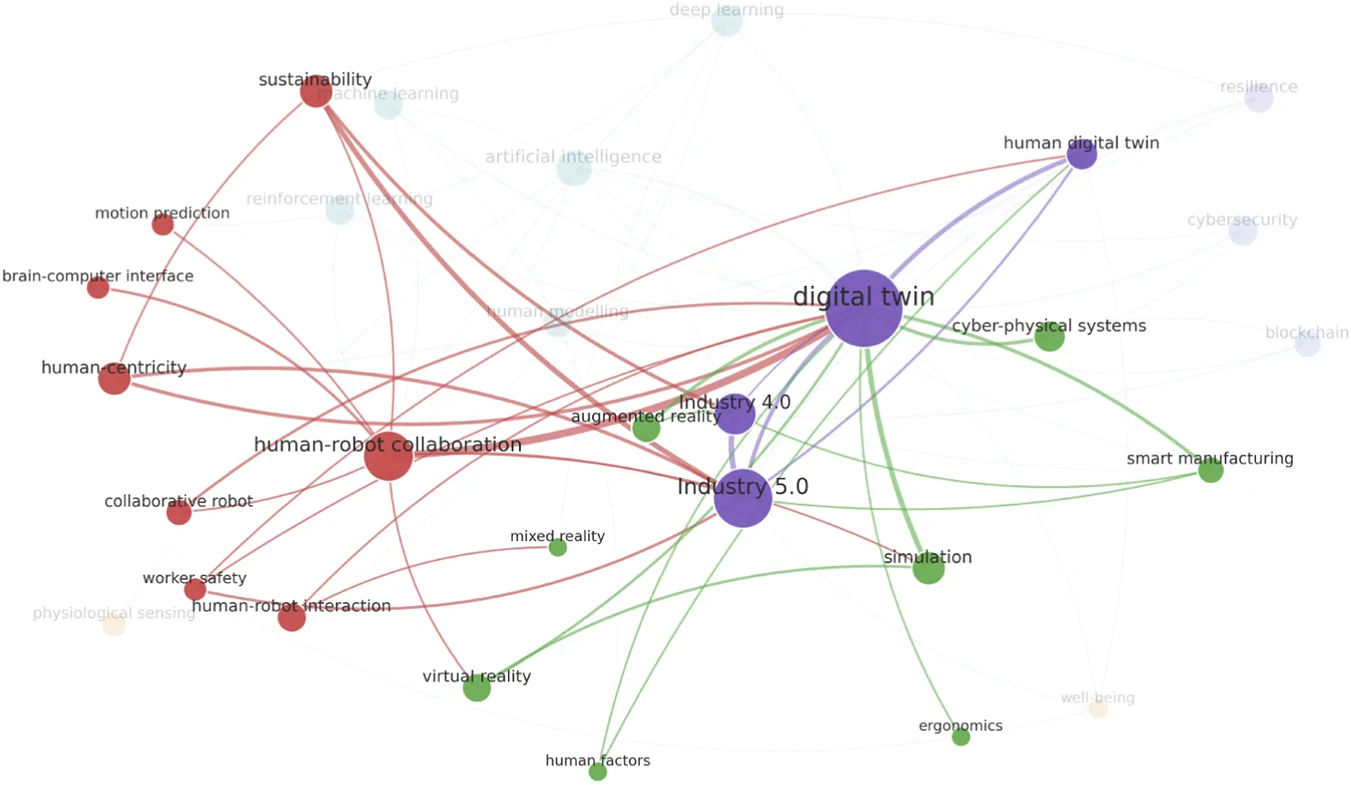
Highlighted title-term co-occurrence network emphasizing the relationships among Digital Twins, Human-Robot Collaboration, Industry 5.0, simulation, worker-centered technologies, and associated human-centric industrial concepts. Secondary terms and connections are faded to improve visual interpretation.

The bibliometric mapping was intentionally restricted to publication titles and was included as a supplementary descriptive analysis. Titles provide a concise indication of the subjects explicitly foregrounded by authors while reducing the influence of generic or incidental terms that may appear in abstracts. The purpose of this analysis was therefore to identify the most visible and explicitly stated themes in the selected literature rather than to provide a comprehensive representation of every concept discussed in the publications. Themes that may not be prominently represented in titles, including ergonomics, worker wellbeing, trust, physiological sensing, fatigue, and intention prediction, were examined through the full-text qualitative synthesis presented in the subsequent sections.

## 3 Concept and definition

I5.0 remains an evolving and contested concept, with no clear consensus regarding its boundaries, defining characteristics, or distinction from I4.0 (Alves et al., 2023). Some definitions emphasize human creativity and collaboration with robots, while others focus on sustainability, resilience, social inclusion, advanced technologies, or worker wellbeing (Madsen and Berg, 2021; Majerník et al., 2022). This diversity indicates that I5.0 currently represents a collection of related technological, human, organizational, and environmental perspectives rather than a uniformly defined industrial paradigm (Sindhwani et al., 2022; ElFar et al., 2021).

Despite these differences, three principles appear consistently across the definitions: human-centricity, sustainability, and resilience (Ivanov 2023; Madsen and Berg, 2021). Human-centric positions workers as active participants whose creativity, experience, judgment, and problem-solving capabilities are supported by intelligent technologies. Sustainability extends industrial objectives towards resource efficiency, environmentally responsible production, and respect for planetary limitations. Resilience refers to the ability of industrial organizations, production systems, and supply chains to adapt to disruptions and maintain or restore essential functions (Perez et al., 2022; Mladineo et al., 2022). Other definitions extend the concept to include human-machine collaboration, mass customization, cognitive systems, and technology-driven innovation (Javaid and Haleem, 2020; Sindhwani et al., 2022; ElFar et al., 2021).

However, these characteristics do not necessarily establish I5.0 as a separate industrial revolution. Unlike previous industrial revolutions, which were associated with identifiable technological discontinuities such as steam power, electricity, digital automation, and interconnected cyber-physical systems, I5.0 does not introduce an equivalently distinct technological foundation. Most of its enabling technologies and underlying concerns were already present in I4.0. I5.0 can therefore be interpreted as an incremental, socio-technical, and value-oriented development of I4.0 rather than its technological replacement (Johri et al., 2021). Its principal contribution lies in redefining the objectives and conditions under which advanced industrial technologies are developed and applied.

Table 2 compares representative definitions identified in the literature. Although their terminology and emphasis differ, the definitions converge around worker centrality, human-machine collaboration, sustainability, resilience, intelligent technologies, and socially responsible production. However, they generally describe the desired characteristics of I5.0 without clearly explaining how these principles can be operationally integrated within a real manufacturing system. Table 3 summarizes the technological and socio-technical evolution from Industry 1.0 to I5.0.

**TABLE 2:** Industry 5.0 concepts and the digital-twin-oriented synthesis developed in this review.

Source	Concise conceptualization	Primary emphasis
Madsen and Berg (2021)	Industry 5.0 seeks to establish sustainable, human-centric, and resilient industrial systems	Three foundational pillars
Majerník et al. (2022)	Industry 5.0 promotes environmentally sustainable, economically resilient, and socially inclusive production centered on workers and human–machine cooperation	Inclusive and socially responsible manufacturing
Ivanov (2023)	Industry 5.0 is founded on the three interdependent principles of human-centricity, sustainability, and resilience	Conceptual foundations
Sindhwani et al. (2022)	Industry 5.0 combines sustainable production, advanced digital systems, and human–robot collaboration to improve workplace and service conditions	Sustainable human–robot collaboration
Javaid and Haleem (2020)	Industry 5.0 integrates emerging and advanced technologies to support industrial innovation	Technology-driven innovation
ElFar et al. (2021)	Industry 5.0 reconciles human and machine capabilities to improve manufacturing capacity and performance	Human-machine complementarity
Maddikunta et al. (2022)	Industry 5.0 combines human creativity with intelligent machines to improve resource efficiency and enable personalized production	Human expertise and production personalization
Alves et al. (2023)	Industry 5.0 places workers and their wellbeing at the center of sustainable and resilient production systems	Worker-centered production
Present review	Industry 5.0 is a human-centric, sustainable, and resilient manufacturing paradigm in which DTs connect humans, robots, AI, and production systems to support real-time, adaptive, and worker-centered decision-making	Digital-twin-enabled human-centric manufacturing

The final row presents the literature-derived, DT-oriented synthesis adopted in this review.

**TABLE 3:** Evolution of industrial revolutions.

Revolution	Main technologies	Industrial and social transformation
Industry 1.0	Steam engines, mechanized textile machinery, and iron manufacturing	Factory system, standardized work, urbanization, division of labour, and hierarchical supervision Sharma and Singh (2020)
Industry 2.0	Electricity, oil, internal-combustion engines, chemical processes, telegraph, and telephone	Mass production, assembly lines, specialized departments, and machine-centered labour Sharma and Singh (2020)
Industry 3.0	Computers, digital electronics, automation systems, and the internet	Flexible production, global supply chains, information-based work, and networked economies Sharma and Singh (2020)
Industry 4.0	Cyber-physical systems, IoT, AI, big-data analytics, additive manufacturing, and advanced robotics	Smart factories, real-time decision-making, predictive maintenance, workforce re-skilling, and cybersecurity concerns Kayikci et al. (2022); Callaghan (2020); Löffler and Tschiesner (2013); Givehchi et al. (2013); Bai et al. (2020); Furstenau et al. (2020)
Industry 5.0	Human-robot collaboration, collaborative robots, DT, AI, IoT, and human-centric intelligent systems	Human-centric, sustainable, and resilient production; worker wellbeing; human–machine collaboration; adaptive workplaces; and responsible use of industrial technologies Zafar et al. (2024); European Commission (2021); Kralj and Aralica (2023); Wang et al. (2024a); Wang et al. (2024b); Davila-Gonzalez and Martin (2024)

The operational criteria presented in Table 4 were synthesized from the characteristics identified in the literature (Baratta et al., 2024; Krupáš et al., 2024; Fontes et al., 2024; Nie et al., 2026; He et al., 2024). An implementation is classified as an HCDT only when all criteria are satisfied. Each criterion is necessary, while their joint satisfaction is sufficient for classification within the scope of this review. These criteria constitute a literature-derived synthesis and have not yet been validated as an industry standard.

**TABLE 4:** Operational criteria for classifying a human-centric digital twin (HCDT).

Criterion	Operational requirement
Worker-specific representation	The system maintains a digital representation of an individual worker and updates it using physical-worker data at a frequency appropriate to the supported decision
Industrial context integration	Human information is integrated with relevant robot, machine, task, process, or environmental data rather than being analyzed in isolation
Closed-loop decision linkage	Human and operational information influences a recommendation, decision, or physical adaptation, and the resulting effects are returned through system or worker feedback
Measurable worker benefit	The system defines and evaluates at least one worker-centered outcome related to safety, ergonomics, wellbeing, autonomy, accessibility, participation, or skill development rather than exclusively targeting productivity
Human authority and data governance	Workers retain appropriate rights to understand, correct, question, reject, or override consequential decisions, with defined provisions for privacy, consent, data access, and data use
Trustworthy and safe operation	The system communicates relevant uncertainty and includes defined validity limits, supervisory controls, and safe or degraded operation when sensing, communication, or model confidence is insufficient

## 4 Human-centric digital twins vs. human-robot collaboration vs. human models

The HCDT, HRC, and human models address the involvement of workers in intelligent manufacturing from different but complementary perspectives. A human model represents selected characteristics of a worker, HRC describes the physical or cognitive interaction between humans and robots, and an HCDT integrates human and industrial information to support adaptive and human-oriented decision-making (He et al., 2024; Krupáš et al., 2024; Nie et al., 2026). Distinguishing these concepts is necessary because the inclusion of a human model or an HRC application does not, by itself, make an industrial system human-centric.

A human model is a digital representation of selected human characteristics. Depending on its purpose, it may include anthropometric dimensions, body posture, movement, muscular load, physiological condition, cognitive workload, behaviour, or emotional state. Human models are commonly used for ergonomic assessment, workplace design, motion analysis, task simulation, and safety evaluation (He et al., 2024). However, many human models are generic, static, or used offline. They may represent a population percentile or a predefined worker profile rather than a continuously updated representation of a specific individual. Consequently, a conventional human model lacks the persistent physical-virtual connection, real-time updating, and personalization normally associated with a HDT (He et al., 2024; Fontes et al., 2024).

Human-Robot Collaboration concerns the organization and execution of tasks in which humans and robots operate within a shared process or workspace. Its purpose is to combine complementary capabilities: humans provide creativity, contextual judgment, dexterity, adaptability, and problem-solving, whereas robots provide precision, repeatability, strength, and consistency. HRC research therefore concentrates on task allocation, shared control, intention recognition, collision avoidance, motion planning, ergonomic improvement, and safe physical interaction (Baratta et al., 2024; Nie et al., 2026). HRC nevertheless describes an interaction arrangement rather than a virtual representation. An HRC system may operate through predefined safety rules and fixed robot behaviour without using either a HDT or an HCDT (Baratta et al., 2024).

A human model can strengthen HRC by enabling the robot or control system to estimate worker posture, reach, motion, fatigue, workload, or intended actions. However, if the model is static or updated only during offline simulation, the collaborative system cannot respond effectively to changes in the worker or workplace. A HDT addresses this limitation by maintaining a personalized and dynamically updated representation of a particular worker (He et al., 2024). Sensor and interaction data can be used to represent physical, physiological, cognitive, behavioral, and emotional states, allowing the system to identify changes in worker capability, fatigue, stress, ergonomic exposure, and task performance (Krupáš et al., 2024; Alfaro-Viquez et al., 2026). The distinction between a human model and a HDT therefore lies not only in the level of representation but also in individualization, continuous data exchange, contextual updating, and feedback to the physical system.

An HCDT extends beyond the digital representation of the worker. It connects human data with information from robots, machines, tasks, processes, and the working environment to create an integrated human-cyber-physical representation (Krupáš et al., 2024; Alfaro-Viquez et al., 2026). This representation can evaluate production requirements and human conditions before recommending or applying an adaptation. In an HRC application, for example, an HCDT may modify robot speed, trajectory, separation distance, assistance level, interface information, or task allocation according to the worker’s position, experience, workload, fatigue, and physiological condition (Cimino et al., 2024; Liao and Cai, 2024; Nie et al., 2026). It can therefore transform HRC from a predominantly reactive and rule-based interaction into predictive, personalized, and context-aware collaboration.

The presence of adaptation alone, however, does not make a system human-centric. A system could use physiological and behavioral data exclusively to increase worker output or reduce production delays. Such an application may contain a HDT but remain production-centered. For an HCDT to be genuinely human-centric, human information must influence decisions that protect or improve safety, ergonomics, wellbeing, autonomy, capabilities, and meaningful participation (Krupáš et al., 2024; Fontes et al., 2024). The worker should also retain appropriate control over personal data and consequential system decisions. This requirement introduces ethical and governance concerns involving privacy, informed consent, data ownership, algorithmic transparency, bias, cybersecurity, human oversight, and the right to withdraw or restrict the use of personal data (Fontes et al., 2024).

The three concepts consequently form a functional relationship rather than a hierarchy of interchangeable terms. The human model provides a representation of relevant human characteristics; HRC provides the operational setting in which human and robot capabilities are combined; and the HCDT provides the dynamic integration and decision-support mechanism needed to make that collaboration adaptive and human-centered. An HCDT may therefore support HRC, but not every HCDT requires a collaborative robot, and not every HRC system qualifies as an HCDT (Baratta et al., 2024; Krupáš et al., 2024; Nie et al., 2026). Accordingly, this review considers a system to be human-centric only when human data affect industrial decisions in ways that provide explicit and demonstrable benefits to the worker, rather than treating the worker merely as another monitored production resource.

### 4.1 Human-centric digital twins: a patient-to-worker case study

The healthcare system discussed in this section is not presented as a validated industrial HCDT implementation. Instead, it provides a conceptual cross-domain transfer example demonstrating how selected human-centric principles from patient monitoring could inform the development of worker-centered DT in I5.0. The authors previously developed a patient-monitoring HDT, as reported in Khan (2024). The system uses unobtrusive ESP32 Wi-Fi and radio-frequency sensors to measure respiration rate and exhaled-hydration characteristics without requiring the person to wear monitoring devices (Khan et al., 2022; 2026a). Signal-processing techniques are used to derive physiological parameters, while ML and DL algorithms classify respiratory conditions and support clinical decision-making. Its relevance to human-centered design lies in its unobtrusive data acquisition and intended support for early detection (Anwar et al., 2024), continuous monitoring, and personalized care. However, these healthcare-domain results do not constitute evidence of effectiveness in an industrial workplace.

At a conceptual level, selected components of this healthcare architecture could be transferred to an I5.0 work environment. The following industrial adaptation is therefore a proposed research direction rather than an implemented or validated industrial system. In the proposed industrial application, the patient is replaced by an industrial worker and unobtrusive sensing is extended to detect physiological and occupational state indicators, including respiration, exertion (Khan et al., 2026b), fatigue, heat strain and hazardous environmental conditions. The physiological measurements of the worker can be coupled with task and machine data and environmental data to ensure an up-to-date digital version of the human workplace system that is constantly updated. The outcome of the HCDT can help to identify potentially unsafe conditions and recommendations for actions to take, such as initiating a rest period, reducing physical demands of a task, modifying a human-machine task allocation, adjust environmental controls, or alerting the worker and safety supervisor.

The AI outputs in this proposed adaptation should be used to supplement, not supplant, human judgment. Any recommendations for interventions should be accepted, rejected or changed at the discretion of workers or authorized persons. Furthermore, physiological parameters should not only be used to boost production, work load or for assessing personal performance. The main goal of gathering and studying these data is to create real measurable enhancements in the safety, ergonomics, health and wellbeing of workers. Informed consent, data minimization, privacy protection, secure access control, explainability of the algorithms and communication of prediction uncertainty are therefore fundamental requirements of the proposed HCDT.

The healthcare implementation provides evidence only for the original patient-monitoring architecture, including unobtrusive sensing, signal processing, physiological modelling, and AI-assisted decision support. Its extension to worker monitoring remains a conceptual cross-domain proposal and should not be interpreted as validated industrial evidence. An operational industrial HCDT would require real-time integration with workplace machines, robots, task-management systems, and environmental sensors; evaluation involving representative workers and operating conditions; and validation of the complete two-way feedback loop between HCDT recommendations, workplace interventions, and worker outcomes. Figure 3 presents an application-level HCDT derived through the conceptual transfer of the healthcare case study to an industrial human-robot setting. Its novelty lies in integrating unobtrusive worker sensing, digital modelling, AI-assisted adaptation, human oversight, and feedback to support worker wellbeing; the proposed industrial application requires future validation.

**FIGURE 3:**
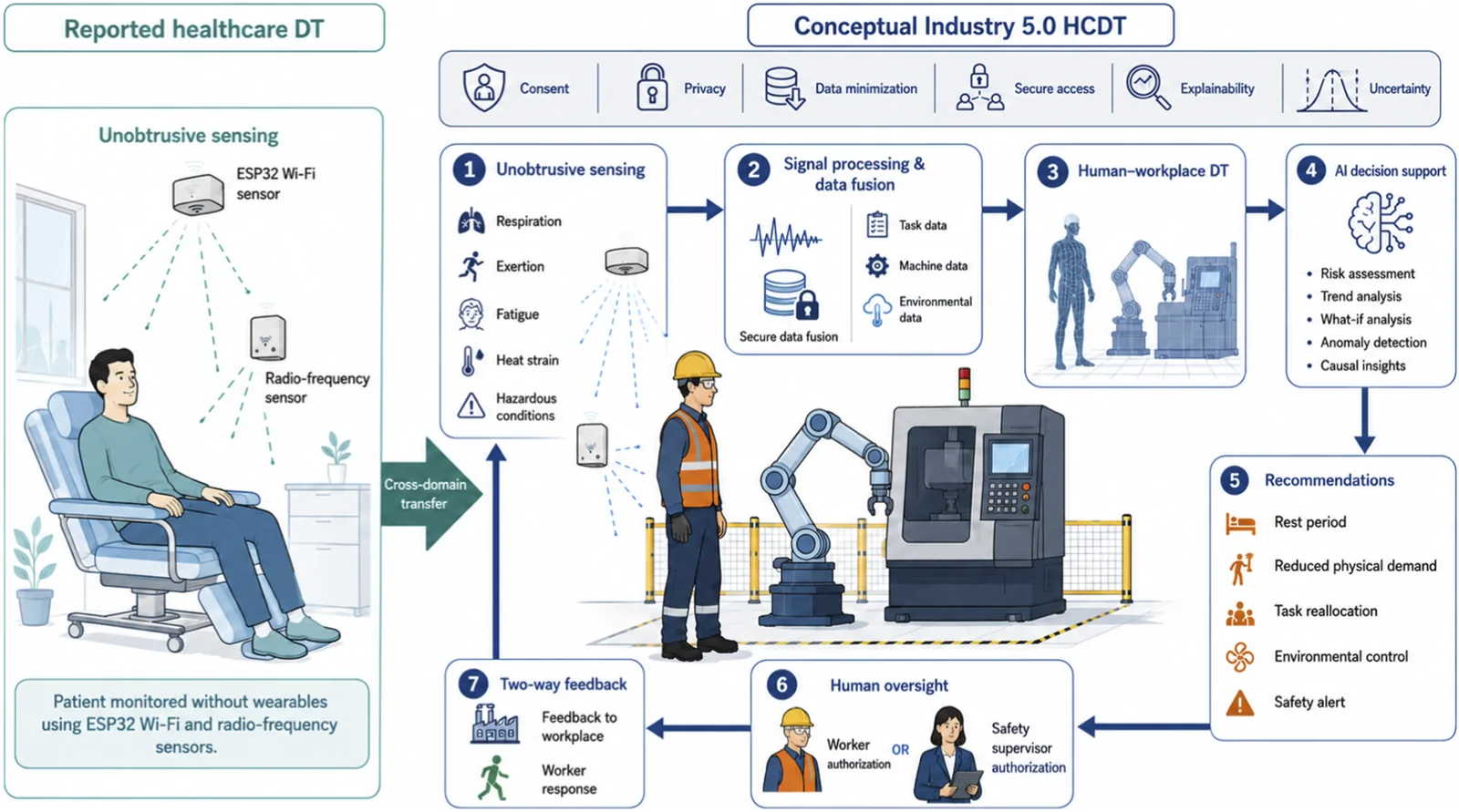
Conceptual cross-domain framework illustrating how unobtrusive healthcare-monitoring principles could be transferred to an I5.0 workplace. The proposed industrial HCDT architecture has not yet been implemented or validated in an operational workplace.

## 5 Enabling technologies

A single technology cannot enable an HCDT. HCDTs emerge from convergence of multi-modal sensing, industrial connectivity, digital modelling, AI, edge–cloud computing, collaboration and human–robot interaction, and secure data-management mechanisms. These technologies cannot be separated, they create a closed loop system in which human, robot, machine, task and environmental data is collected, communicated, analyzed, modeled and represented in the DT, and returned to humans in the form of a recommendation or physical change. I5.0 is not necessarily defined by the novelty of the technologies used but rather their synergy to ensure worker safety, wellbeing, autonomy, sustainability, and industrial resilience. Figure 4 represents the I5.0 pillars and enabling technologies for HCDT.

**FIGURE 4:**
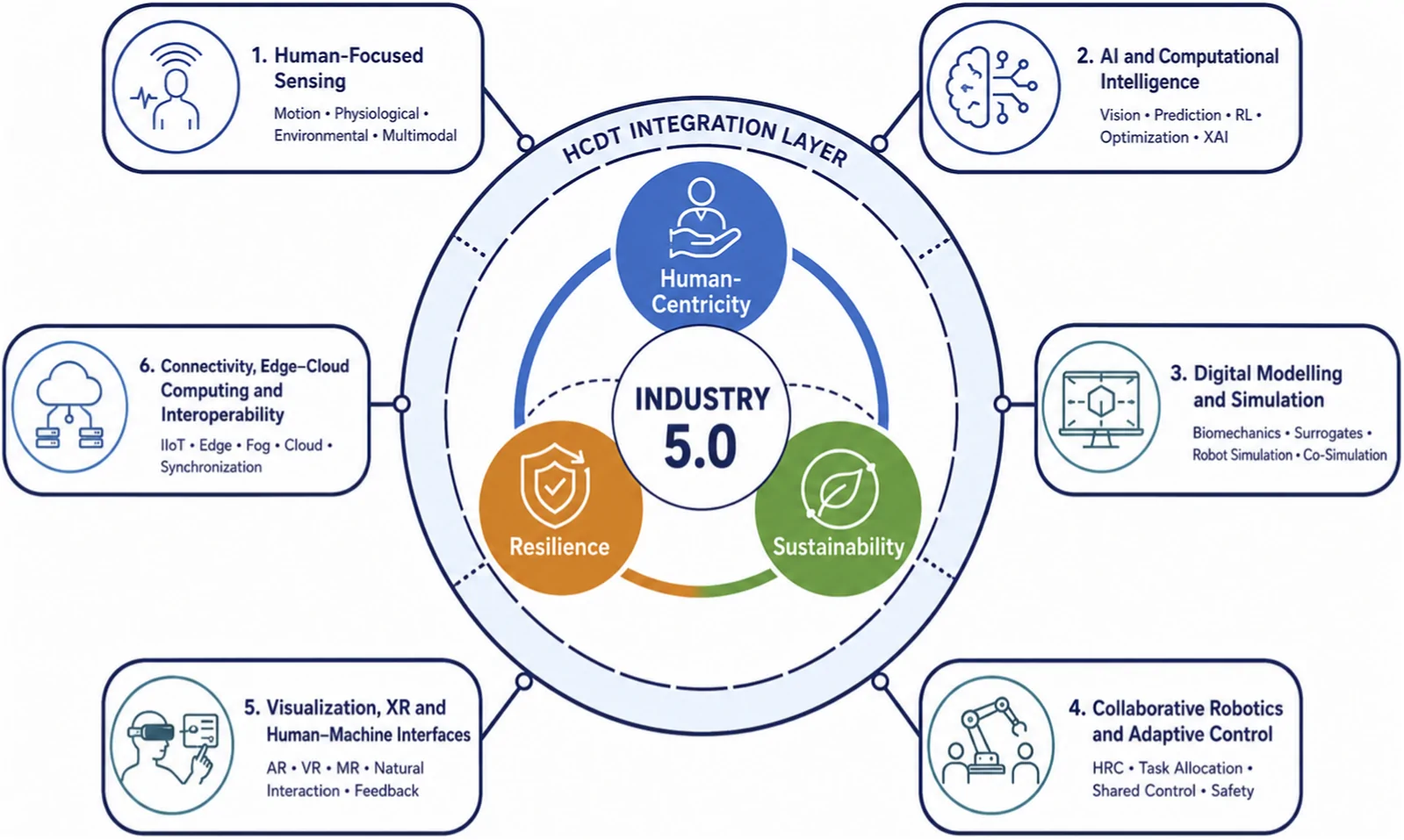
I5.0 pillars and enabling technologies for human-centric, AI-enabled manufacturing. HDT, AI, multi-modal sensing, collaborative robotics, industrial IoT, and edge–cloud systems collectively support human-centricity, resilience, and sustainability.

### 5.1 Human-focused sensing technologies

Sensors provide the physical-to-digital connection required to construct and continuously update a HCDT. Although the acquisition of data from machines, robots, and industrial processes is relatively mature, the reliable representation of human physical, physiological, cognitive, behavioral, and emotional states remains challenging (Asad et al., 2023a). Human-focused sensing technologies can be broadly classified into motion and visual sensors, physiological sensors, environmental sensors, and direct human–machine input devices.

Optical motion-capture systems use active or passive markers to estimate body-segment positions, joint angles, and movement trajectories. These systems can provide high measurement accuracy, but they normally require instrumentation of the worker and a controlled measurement environment. Unmarked approaches are less intrusive and employ RGB, RGB-D, infrared, or depth cameras to estimate human posture and motion. For example, Yi et al. (2022) developed a vision-based human-robot collaboration system in which RGB-D measurements and computer-vision algorithms were used to connect human-posture information with a digital twin. Similarly, Dimitropoulos et al. (2021) combined RGB-D sensing, wearable devices, convolutional neural networks, and synthetic training data to support safe and ergonomically informed collaborative assembly.

Camera-based sensing has also been integrated with digital twins for continuous ergonomic assessment. Greco et al. (2020) developed a digital-twin framework for monitoring workers’ postures during manufacturing operations and identifying ergonomically unsuitable conditions. These approaches allow the HCDT to represent the worker’s posture, movement, proximity to equipment, and interaction with the production environment. Nevertheless, their performance can be affected by occlusion, illumination, camera placement, restricted fields of view, and privacy requirements.

Wearable inertial measurement units provide an alternative where camera-based systems are unsuitable. Accelerometers, gyroscopes, and magnetometers can measure body-segment orientation, movement, posture, and trajectory without requiring a fixed camera infrastructure. A comprehensive review by Filippeschi et al. (2017) showed that inertial sensing is particularly suitable for tracking upper-limb motion, although measurement accuracy can be affected by sensor drift, magnetic interference, calibration errors, and variations in sensor placement.

Using only six inertial sensors, Yi et al. (2021) demonstrated that deep neural networks and motion constraints could reconstruct three-dimensional human posture and global translation in real time. Wearable inertial sensing can therefore support the development of personalized worker representations in environments containing visual obstructions or large working areas. However, sensor attachment, battery requirements, recalibration, and worker comfort must be considered before continuous industrial deployment.

Physiological sensing extends the HCDT beyond the geometric representation of the human body. Biological signals may provide information about muscular activity, visual attention, cognitive workload, fatigue, stress, and operator intention. Physiological responses have consequently been investigated as inputs for evaluating human conditions during human–robot interaction (Tiberio et al., 2013).

Electrooculography (EOG) measures electrical activity associated with eye movement and can support gaze-related human–computer interaction (Usakli et al., 2010). Electroencephalography (EEG) records electrical activity from the scalp and has been investigated for brain-controlled robotic systems and Brain–Computer Interfaces (BCIs) (Bi et al., 2013). Electromyography (EMG), in contrast, measures muscle activation and can support the recognition of movement intention and physical effort (Hakonen et al., 2015). Other measurements, including heart rate, heart-rate variability, electrodermal activity, respiration, and skin temperature, may contribute to the estimation of stress, fatigue, cognitive demand, and emotional condition.

Brain–Computer Interfaces represent a more direct, although still emerging, form of communication between workers and industrial systems. Dmytriyev et al. (2022) demonstrated an EEG-based interface through which an operator could issue commands during a human-robot collaborative assembly task. Ji et al. (2021) subsequently integrated a BCI with augmented-reality feedback to create a closed-loop interaction mechanism for industrial human-robot collaboration. These technologies could enable the HCDT to receive explicit or implicit information about worker intention. However, physiological variability, signal noise, lengthy calibration, limited classification accuracy, and cognitive burden currently constrain their widespread industrial use.

Environmental sensors provide the contextual data required to interpret human measurements correctly. Temperature, humidity, airflow, illumination, noise, vibration, dust, ultraviolet radiation, and hazardous-gas measurements can be incorporated into the HCDT to evaluate occupational exposure, comfort, and environmental risk. Human measurements should not be interpreted independently of these conditions. For instance, a change in heart rate may be caused by physical effort, psychological stress, thermal exposure, or normal body movement. Multi-modal fusion of physiological, motion, task, and environmental data is therefore needed to determine the most probable cause of a measured change (Asad et al., 2023a).

The selection of sensing technologies should be determined by the intended HCDT function rather than by the availability of sensors. Safety-critical applications require sufficient accuracy, reliability, update frequency, and fault detection, whereas worker-centered applications additionally require comfort, proportionality, informed consent, and data minimization. Only measurements necessary for a defined worker or operational benefit should be acquired. Sensor uncertainty and missing data should also be represented explicitly so that the HCDT does not treat an incomplete or unreliable measurement as an accurate description of the worker.

### 5.2 Artificial intelligence and computational intelligence

Artificial intelligence (AI) and computational-intelligence methods transform the heterogeneous data collected from workers, robots, machines, tasks, and industrial environments into information that can be used by a HCDT. Their functions include human-pose estimation, activity recognition, intention prediction, fatigue and workload assessment, anomaly detection, motion planning, task allocation, and adaptive robot control. AI therefore forms the analytical connection between sensing the human-cyber-physical system and selecting an appropriate operational response (Asad et al., 2023a; Huang et al., 2021).

#### 5.2.1 Computer vision

Computer vision is required when an HCDT must recognize human posture, movement, gestures, facial expressions, tools, personal protective equipment, and surrounding objects. Deep Neural Networks (DNN), particularly Convolutional Neural Networks (CNNs), can extract spatial features from RGB, RGB-D, infrared, and depth images. These features can be used to estimate the position and configuration of workers and robots within a shared workspace.

For example, Yi et al. (2022) used RGB-D measurements and a CNN-based approach to estimate human posture in a vision-based human-robot collaborative digital-twin system. Dimitropoulos et al. (2021) combined computer vision, wearable devices, and AI to support object recognition, human-pose estimation, and ergonomically informed collaborative assembly. Their system used both real and synthetically generated images for model training, demonstrating that virtual environments can help generate labeled data when the collection of real industrial images is difficult or hazardous.

ML-enhanced DTs have also been developed for monitoring human-robot collaboration. Dröder et al. (2018) connected machine-learning-based human-motion recognition with a digital representation of a collaborative workstation. Similarly, Islam et al. (2019) used visual information to support safe and flexible cyber-physical production. These systems allow the HCDT to detect worker location and actions, estimate the possibility of physical conflict, and update robot behaviour accordingly.

However, computer-vision accuracy can deteriorate because of occlusion, changing illumination, reflective surfaces, unusual worker postures, motion blur, or differences between training and deployment environments. A prediction should therefore be accompanied by an uncertainty or confidence estimate. Safety-critical decisions should not depend exclusively on an unverified vision model, particularly when the worker is partially outside the camera field of view.

#### 5.2.2 Human-motion and intention prediction

An HCDT should not only represent the worker’s current state but also estimate how the worker and production system are likely to evolve. Human-motion prediction allows collaborative robots to begin adjusting their speed or trajectory before a worker enters a hazardous area. Intention prediction can similarly identify whether a worker intends to request assistance, transfer an object, interrupt a task, or enter the robot’s workspace.

Supervised learning methods used for these purposes include CNNs, Long Short-Term Memory (LSTM) networks, Hidden Markov Models (HMMs), and Spiking Neural Networks (SNNs). Because human activity develops over time, recurrent and temporal models are generally more suitable than models that analyze each measurement independently.

Liu et al. (2019) developed a context-aware deep-learning approach for predicting human movement during collaborative manufacturing. The approach considered both observed worker motion and the surrounding operational context. Zhang et al. (2022) proposed a multi-modal fusion-based SNN for predicting when a worker would request robot collaboration. These studies demonstrate that combining motion, task, and contextual information can enable proactive rather than exclusively reactive human–robot collaboration.

Nevertheless, human behaviour is inherently variable and cannot always be predicted reliably. The HCDT should therefore treat predicted intention as a probabilistic estimate rather than an established fact. Robot adaptation should be proportional to prediction confidence, and uncertainty should cause the system to move towards a safer operating state instead of executing an aggressive production action.

#### 5.2.3 Reinforcement learning

Reinforcement Learning (RL) enables an agent to learn a decision or control policy by interacting with an environment and receiving rewards or penalties. When combined with DTs, RL policies can be trained and evaluated in a virtual environment before they are transferred to a physical robot or manufacturing system. This reduces the cost, disruption, and physical risk associated with direct experimentation in a real collaborative workplace.

Digital-twin-supported RL has been investigated for robot grasping, motion planning, localization, task scheduling, collaborative assembly, and manufacturing optimization. Xia et al. (2021) developed a digital-twin environment for training deep-RL agents for smart manufacturing. Their framework integrated the simulated environment, communication interfaces, and learning intelligence needed to train and evaluate decision policies. Matulis and Harvey (2021) similarly used a digital twin and RL to train the control of an industrial robot arm.

In human-robot collaboration, the reward function must include worker-oriented criteria. Liu et al. (2021) developed a deep-RL approach for safe industrial human-robot interaction using an intrinsic reward function. Such reward functions may consider collision avoidance, separation distance, task completion, robot smoothness, human waiting time, and ergonomic exposure. If the reward function includes only production rate or cycle time, the learned policy may achieve high productivity while creating excessive physical or cognitive demands for the worker.

The main technical limitation is the difference between the simulated and physical systems, commonly described as the simulation-to-reality gap. A policy that performs well in the DT may fail when exposed to sensor noise, communication delays, unexpected human behaviour, or inaccurate physical parameters. Before physical deployment, RL policies should therefore be tested under uncertainty, constrained by verified safety rules, and monitored by an independent safety controller.

#### 5.2.4 Optimization techniques

Optimization techniques support the selection of robot trajectories, workstation layouts, production schedules, human-robot task allocations, and operating parameters. Depending on the complexity of the problem, HCDTs can employ mathematical programming, simulated annealing, genetic algorithms, particle-swarm optimization, ant-colony optimization, or multi-objective evolutionary methods.

Nourmohammadi et al. (2022) used optimization to balance and schedule assembly lines containing both human and robot tasks. Bansal et al. (2019) applied ant-colony optimization to industrial robot programming within a DT environment. These approaches demonstrate how computational search can evaluate multiple operational configurations before the selected configuration is implemented physically.

For Industry 5.0 applications, the optimization objective should extend beyond production cost and cycle time. Worker safety, muscular loading, cognitive workload, autonomy, energy consumption, production resilience, and environmental impact should be included as either objectives or constraints. Because these objectives may conflict, the HCDT should present the resulting trade-offs rather than conceal them within a single aggregate performance score.

#### 5.2.5 Hybrid and explainable intelligence

Although data-driven AI can identify complex patterns, model-based control remains important for safety-critical human-robot collaboration. Analytical and physics-based models provide information about system structure and can offer stability, performance, or safety guarantees that are difficult to obtain from an unconstrained neural network. Mujica et al. (2023) demonstrated robust variable-admittance control for human-robot co-manipulation when the manipulated load was unknown.

A practical HCDT should therefore combine data-driven and model-based intelligence. Machine-learning models can estimate worker state, intention, or uncertain system characteristics, while verified controllers and physical constraints determine which actions are permissible. This hybrid structure can improve adaptability without removing established safety guarantees.

Explainability is equally important because workers and supervisors must understand consequential recommendations. When the HCDT recommends reducing robot speed, reallocating a task, or interrupting production, it should identify the measurements and model outputs responsible for that recommendation. The system should communicate prediction confidence, relevant uncertainty, and the expected consequences of accepting or rejecting the proposed action.

AI should consequently operate as decision support rather than an unquestioned replacement for human judgment. Workers should retain the ability to correct an inaccurate worker-state estimate, reject an unsuitable adaptation, and regain control when automated behaviour becomes confusing or unsafe. These requirements distinguish human-centric computational intelligence from production-centered automation.

### 5.3 Digital modelling and simulation technologies

Digital modelling and simulation constitute the analytical core of a HCDT. While sensing technologies provide observations of the worker and industrial environment, simulation models represent the relationships among workers, robots, machines, tasks, and production processes. These models allow alternative operating conditions to be evaluated before physical changes are applied, thereby reducing the cost and safety risks associated with direct experimentation (Asad et al., 2023a).

Depending on the application, an HCDT may integrate geometric human models, biomechanical models, finite-element models, robot simulations, discrete-event production models, data-driven surrogate models, and immersive virtual environments. The appropriate modelling approach is determined by the physical phenomenon being represented, the required prediction accuracy, the available data, and the computational time allowed for decision-making.

#### 5.3.1 Numerical and biomechanical modelling

Numerical modelling can represent physical phenomena that cannot be measured or tested safely during normal industrial operation. Finite-Element Analysis (FEA), for example, can simulate structural deformation, mechanical stress, contact forces, fluid flow, heat transfer, and biomechanical responses. Commercial platforms including ANSYS, ABAQUS, and COMSOL are frequently used to construct high-fidelity models of physical assets and human-related systems.

In HCDT applications, biomechanical models may be used to estimate joint loading, muscular demand, collision consequences, repetitive-strain exposure, and the physical effects of human-robot contact. Asad et al. (2023b) developed computational models of human-robot accident scenarios involving high-payload industrial robots. Their study showed how biomechanical simulation could estimate the severity of contact events without exposing human participants to hazardous experimental conditions.

Similar approaches have been used in healthcare to construct patient-specific DTs. Although the clinical and industrial objectives are different, these studies demonstrate how individual geometry, material properties, boundary conditions, and measured data can be integrated to create personalized physical models. Such principles can be transferred to worker-centered applications involving ergonomics, assistive devices, exoskeletons, workplace design, and human–robot safety.

However, a detailed biomechanical representation does not automatically constitute an HCDT. A static model used only during workstation design remains a human model. To function as part of an HCDT, the model should be personalized, updated using information from the physical worker, and connected to an operational decision or adaptation.

#### 5.3.2 Reduced-order and surrogate models

High-fidelity numerical simulations are generally computationally expensive. A detailed finite-element model may require several minutes or hours to evaluate one operating condition, making it unsuitable for real-time robot control or continuous worker-state assessment. Reduced-Order Models (ROMs) and data-driven surrogate models address this limitation by approximating the response of a high-fidelity simulation using a less computationally demanding representation.

Kapteyn (2021) showed that reduced-order modelling provides an important mathematical foundation for developing predictive DTs at scale. Instead of executing the complete physical model whenever new data arrive, a reduced representation can estimate the most relevant system behaviour with substantially lower computational cost.

Machine learning can also be used to construct surrogate models from high-fidelity simulation data. Liang et al. (2018) demonstrated that deep learning could estimate stress distributions as a faster surrogate for finite-element analysis. Calka et al. (2021) similarly applied machine-learning-based model-order reduction to a biomechanical model. These approaches indicate that an HCDT can use detailed offline simulations to generate training data and then deploy a lightweight surrogate model for real-time operation.

The use of a reduced model introduces a trade-off between computational speed and physical accuracy. The HCDT should therefore identify the operating range within which the surrogate model has been validated. If sensor measurements move outside this range, the system should not continue treating the surrogate prediction as reliable. A higher-fidelity model, model recalibration, or human review may then be required.

#### 5.3.3 Robot simulation platforms

Robot simulators allow researchers and engineers to design, test, and evaluate robotic systems in a virtual environment before deploying them in a shared human workspace. Simulation can be used for motion planning, collision checking, control-system development, sensor evaluation, robot programming, workspace design, and safety analysis.

The Robot Operating System (ROS) and ROS2 provide modular software architectures for connecting sensing, communication, planning, and control components. ROS2 extends this approach with improved support for distributed systems, real-time operation, security, and industrial deployment (Macenski et al., 2022). Gazebo is commonly integrated with ROS or ROS2 to simulate robots, sensors, and physical environments. MoveIt can provide manipulation functions including inverse kinematics, motion planning, collision detection, trajectory generation, and robot-state visualization.

Kousi et al. (2021) combined ROS, Gazebo, MoveIt, and production-system simulation to develop a DT for designing and reconfiguring human-robot collaborative assembly lines. This type of co-simulation allows robot behaviour to be assessed together with task sequences, workstation configuration, human participation, and production performance.

Other robotic simulation platforms include CoppeliaSim, Webots, MORSE, MATLAB/Simulink, and NVIDIA Isaac Sim. Farley et al. (2022) compared the motion accuracy of several mobile-robot simulators and demonstrated that simulator selection can materially affect experimental results. Rojas et al. (2022) developed a reinforcement-learning library for mobile robots using NVIDIA Isaac Sim, illustrating the use of GPU-accelerated simulation for training AI-controlled robotic systems.

A simulator should therefore not be selected only because of software availability. Relevant criteria include physical accuracy, numerical stability, supported robots and sensors, communication interfaces, real-time performance, interoperability, licensing, and the ability to connect the simulation with the physical system.

#### 5.3.4 Game engines and immersive simulation

Game engines provide another technological foundation for HCDTs. Unity and Unreal Engine combine real-time rendering, physics simulation, user interaction, animation, networking, and support for virtual- and augmented-reality devices. These capabilities make them suitable for constructing interactive virtual workplaces and visualizing the relationships among workers, robots, machines, and products.

Kuts et al. (2019) used the Unity game engine to develop a synchronized digital twin and virtual-reality representation of an industrial robotic cell. The virtual robot was connected with its physical counterpart, enabling synchronized control and simulation. Game engines have also been used to evaluate human–robot workspaces, create synthetic computer-vision data, develop immersive training environments, and examine alternative production layouts.

The graphical realism of a game engine should not, however, be confused with physical validity. A photorealistic robot or human model may still contain inaccurate mass, friction, contact, joint, or control parameters. If the simulation is used only for visualization, this limitation may be acceptable. 1f it is used to predict collision forces, ergonomic loads, or safety-critical robot behaviour, its physical assumptions must be verified experimentally.

#### 5.3.5 Co-simulation and model integration

A complete HCDT may require several simulation environments because no individual tool can represent all relevant human and industrial phenomena. For example, a biomechanical model may represent the worker, a robot simulator may model the cobot and its controller, and a discrete-event model may represent production flow and task allocation. Co-simulation enables these models to exchange data while maintaining their specialized functions.

The models may operate at different temporal scales. Robot control and collision monitoring require high-frequency updates, whereas ergonomic exposure, worker fatigue, production scheduling, and sustainability may be evaluated over longer intervals. The HCDT architecture must therefore define how frequently each model is updated, which variables are exchanged, how time synchronization is maintained, and how inconsistencies between model outputs are resolved.

Co-simulation also supports multi-objective evaluation. Before changing a task allocation, the HCDT could estimate its expected effects on cycle time, worker workload, collision risk, energy consumption, and system resilience. This capability is important because an adaptation that improves one Industry 5.0 objective may negatively affect another.

#### 5.3.6 Model verification, validation, and updating

The usefulness of an HCDT depends on the validity of its models. Verification determines whether the model has been implemented correctly, while validation determines whether it represents the physical worker and industrial system with sufficient accuracy for its intended use. These processes should evaluate model assumptions, parameters, boundary conditions, input uncertainty, and sensitivity to measurement errors.

Human-related models require particular attention because worker characteristics vary between individuals and change over time. Strength, movement patterns, reaction time, fatigue, experience, injury, and cognitive condition cannot be represented accurately using a fixed generic model. An HCDT must therefore be initialized using appropriate worker information and re-calibrated when new measurements indicate that its representation is no longer accurate.

Model fidelity should be matched to the supported decision. High-fidelity simulations may be required for offline collision or biomechanical analysis, while simplified models may be necessary for real-time adaptation. A multi-fidelity architecture can combine these requirements by using lightweight models during operation and detailed models for periodic evaluation and recalibration.

The HCDT should also communicate model uncertainty. A recommendation should not be presented as certain when it is based on incomplete sensor data, an unvalidated operating condition, or a simplified model. Confidence measures, validity boundaries, and fallback procedures are therefore necessary before simulation outputs are used to modify physical human-robot operations.

### 5.4 Collaborative robotics and adaptive control

Collaborative robots, commonly known as cobots, provide the principal physical mechanism through which decisions generated by a HCDT can modify manufacturing operations. Unlike conventional industrial robots that generally operate within physically separated workspaces, cobots are designed to share tasks and, under controlled conditions, workspaces with human operators. Their purpose within I5.0 is to complement human capabilities rather than remove workers from the production process. Robotic-arm control consists of a series of sequential tasks: kinematic analysis, path planning, trajectory optimization, control selection, and more recently, Brain–Computer Interaction (BCI) Elgohr et al. (2025c). These capabilities enable realization of HCDT-induced adaptations, but their connection to worker models, human supervision, and safety-related functions is essential to realize human-centered industrial operation.

Humans provide creativity, contextual judgment, adaptability, dexterity, and experience, whereas robots provide precision, repeatability, strength, and consistent execution. The HCDT connects these complementary capabilities by continuously representing the worker, robot, task, and working environment. Based on this representation, it can recommend or implement adaptations to robot speed, trajectory, force, separation distance, assistance level, and task allocation (Baratta et al., 2024).

#### 5.4.1 HCDT-enabled human–robot collaboration

Conventional Human-Robot Collaboration (HRC) is often implemented using predefined robot trajectories, fixed task sequences, and rule-based safety mechanisms. Such systems can react to a worker entering a monitored area but may not account for differences in worker experience, physical capability, fatigue, workload, or intention. An HCDT extends HRC by maintaining an individualized and dynamically updated representation of the worker and production context.

Maruyama et al. (2021) developed a DT-driven HRC approach using a digital human representation. The system connected information from the physical worker and robot with their virtual counterparts to support the planning and evaluation of collaborative operations. Similarly, Baratta et al. (2024) identified digital twins as an important mechanism for integrating simulation, human information, robot data, and adaptive decision-making in collaborative manufacturing.

HCDT-enabled collaboration can consequently progress through three operational levels. At the first level, the twin monitors the worker and robot without modifying their behaviour. At the second level, it provides recommendations or warnings to the worker or supervisor. At the third level, it automatically adapts robot or production behaviour within predefined safety and authority limits. The selected level should depend on the consequence of an incorrect adaptation and the degree of confidence in the HCDT output.

#### 5.4.2 Adaptive robot speed and trajectory

Worker position and predicted movement can be used to modify the robot’s trajectory and speed. When a worker approaches the collaborative workspace, the HCDT may reduce robot speed, increase separation, select an alternative path, or initiate a protective stop. If the predicted worker movement presents no conflict, the robot may continue operating without an unnecessary interruption. Bidirectional rapidly exploring random trees, metaheuristic optimization, and B-spline smoothing were recently applied in the field of robotic-motion-planning to create collision free and dynamically improved trajectories for the motion of a 6 degree-of-freedom robotic arm Elgohr et al. (2026a). These methods may enable HCDT-based trajectory adaptation, but need to be tested for safety and predictability in common human-robot work spaces.

A DL-enhanced digital-twin framework was developed by Wang S. et al. (2024) to improve safety and reliability in human-robot collaborative manufacturing. The framework used human and robot information to support behaviour monitoring and the maintenance of safe separation. Cimino et al. (2024) similarly demonstrated how simulation-based DT can evaluate and improve collaborative assembly operations before adaptations are introduced into the physical system. The path length and smoothness of six degree of freedom (6DOF) robot arm trajectories have also been improved using a hybrid whale-Genetic Algorithm (GA) approach Elhadidy et al. (2026). While the method shows the possibilities for computational optimization, the approach was tested offline as a trajectory planning technique, and may need to be physically and human–robot tested for its real-time use in HCDTs. The results of comparative assessments of particle swarm optimization and whale optimization have also shown that the optimal choice of the optimization method influences the path length, smoothness and convergence behaviour in terms of collisions Elgohr et al. (2025a). The results are applicable to the computational optimization of robot trajectories, but if the HCDT was to be deployed, real-time performance and worker-safety validation would also need to be performed.

Motion prediction can make these adaptations proactive. However, predicted human trajectories contain uncertainty because workers may unexpectedly stop, change direction, or perform an unobserved action. The robot should therefore maintain a safety margin that reflects prediction confidence. When confidence decreases, the controller should adopt a more conservative speed, trajectory, or separation requirement.

#### 5.4.3 Adaptive task allocation

Task allocation determines how work is divided between the human and robot. Fixed allocation assigns the same activities throughout production, whereas adaptive allocation modifies responsibilities according to the current worker, robot, task, and production state.

An HCDT may assign repetitive, hazardous, physically demanding, or high-precision activities to the robot while retaining tasks requiring judgment, creativity, dexterity, or problem-solving for the worker. The allocation can be modified when the HCDT identifies fatigue, excessive ergonomic exposure, high cognitive workload, equipment degradation, or a change in production demand.

Kousi et al. (2021) used a DT to support the design and reconfiguration of human–robot collaborative assembly lines. Their approach demonstrates how virtual models can evaluate alternative distributions of human and robot tasks before physical reconfiguration. Adaptive allocation should nevertheless avoid using physiological or performance data solely to maximize worker output. Changes should provide an explicit benefit such as reduced ergonomic exposure, improved safety, greater accessibility, or better workload balance.

Workers should also retain an appropriate level of influence over task allocation. A worker may possess contextual knowledge that is unavailable to the HCDT, including an unusual product condition, discomfort, equipment behaviour, or an anticipated process interruption. The interface should therefore allow the worker to accept, reject, or request modification of a proposed allocation.

#### 5.4.4 Shared control and physical assistance

Shared control combines human input with robot assistance. The human may determine the task objective or general direction of movement, while the robot provides force assistance, stabilization, precision, or collision avoidance. This is particularly relevant to co-manipulation tasks in which a worker and robot jointly handle the same object.

Mujica et al. (2023) developed robust variable-admittance control for human–robot co-manipulation under unknown loading conditions. Admittance control allows the robot to modify its motion in response to forces applied by the human operator. When integrated with an HCDT, controller parameters could be adapted according to the worker’s capability, task condition, payload, posture, or estimated fatigue Liu et al. (2025).

Imitation learning and tele-operation can also transfer human skills to robotic systems. Lee et al. (2022) proposed an imitation-learning-based tele-operation framework that connected a human digital twin with a robot digital twin. This approach enabled human demonstrations to inform robot control while maintaining a virtual representation of both participants.

However, adaptive assistance can reduce human situational awareness if the automation performs too much of the task without clearly communicating its actions. The worker should understand when assistance is active, what the robot is controlling, and how manual authority can be recovered.

#### 5.4.5 Safety-rated control

AI-based adaptation must not replace certified robot-safety functions. Collaborative-robot systems require safeguards such as monitored stopping, hand-guiding control, Speed and Separation Monitoring (SSM), and Power and Force Limiting (PFL). These measures restrict robot motion or contact forces to reduce the probability and severity of human injury.

Gualtieri et al. (2022) developed and validated safety guidelines for human–robot collaborative assembly systems. Their work emphasized that safe collaboration depends on the complete system, including the robot, end effector, tool, workpiece, task, workspace, control software, and worker.

The HCDT may strengthen these measures by providing richer and more predictive information. Nevertheless, emergency stopping, safe-speed monitoring, force limitation, and separation control should remain available independently of non-safety-rated AI components. If the HCDT, communication network, or AI model fails, the robot should transition to a predefined safe state.

#### 5.4.6 Human-automation conflict and intervention

Adaptive systems introduce the possibility of conflict between human and automated decisions. The worker and HCDT may interpret the same operational condition differently. The HCDT may, for example, recommend continuing production when the worker believes the task is unsafe, or it may stop the robot because of a falsely detected hazard.

Such conflict is particularly important when AI outputs influence safety-critical control. The system should communicate why an adaptation has occurred, which observations produced the decision, and how confident the model is. Workers should have an accessible mechanism for stopping the robot, rejecting an automated recommendation, correcting an inaccurate HCDT state, and escalating unresolved conflicts.

Human intervention should not be treated as system failure. Instead, it provides information that can be used to update the HCDT and improve future decisions. However, learning from intervention must occur within a controlled model-validation process. A single worker correction should not automatically alter safety-critical behaviour for all workers or operating conditions.

#### 5.4.7 Balancing adaptation and predictability

Personalization can improve collaboration, but excessive or unpredictable adaptation may increase cognitive workload and reduce worker trust. If robot speed, trajectory, or task behaviour changes without a clear explanation, the worker may find it difficult to anticipate robot movement. HCDT adaptation should therefore remain bounded, gradual where possible, and visible to the worker. In recent studies, trust is not only considered as a result of successful interaction, but as a design principle of HRC. Therefore, when the behaviour of the robot changes in response to the human or environmental conditions, there will be a need for a calibrated trust in the robot’s behaviour and that requires explainability, predictability, adaptive control, and appropriate human oversight Elgohr et al. (2026b).

The appropriate objective is not maximum robot autonomy but an effective distribution of authority between the human and automated system. Low-risk and reversible adjustments may be performed automatically, whereas high-consequence decisions should require human confirmation or remain under certified safety control. This graded approach allows HCDTs to provide adaptive assistance while retaining predictability, human oversight, and safe physical operation.

### 5.5 Visualization, extended reality, and human-machine interfaces

Visualization and Human–Machine Interfaces (HMIs) connect the HCDT with workers, engineers, and decision-makers. While sensors transfer information from the physical workplace to the digital representation, interfaces communicate the HCDT state, predictions, warnings, and recommended actions back to the human. They also allow workers to provide instructions, correct inaccurate representations, reject unsuitable adaptations, and regain control of automated operations.

The technologies used for this purpose include three-dimensional modelling, conventional visual displays, Augmented Reality (AR), Virtual Reality (VR), Mixed Reality (MR), gesture recognition, voice interaction, haptic feedback, and natural-language interfaces. Their function is not merely to visualize a digital model but to improve human understanding, participation, and control within the human-cyber-physical system (Asad et al., 2023a).

#### 5.5.1 Three-dimensional modelling and visualization

Three-dimensional models provide the geometric foundation for visualizing workers, robots, machines, products, and industrial environments. Software including Blender, Autodesk 3ds Max, CATIA, and Siemens NX can be used to develop geometric representations, while Unity, Unreal Engine, and NVIDIA Omniverse support real-time rendering and interaction.

A visual HCDT can display the present state of a production system, historical changes, predicted future conditions, and alternative operational scenarios. Information such as robot trajectories, worker posture, hazardous regions, ergonomic loading, task progress, machine condition, and energy consumption can be spatially associated with the relevant physical or virtual object.

The visualization should distinguish among measured, estimated, predicted, and simulated information. A sensor measurement should not be displayed in the same manner as an uncertain AI prediction. Confidence intervals, uncertainty indicators, timestamps, and data-quality warnings can prevent users from interpreting an outdated or uncertain HCDT state as a direct measurement.

Synthetic three-dimensional environments can also support the development of AI systems. Alexopoulos et al. (2020) demonstrated how DT-generated information could support supervised machine-learning applications in manufacturing. Virtual models can generate labelled images of workers, robots, tools, and hazardous situations that would be expensive or unsafe to reproduce physically.

#### 5.5.2 Augmented reality

Augmented Reality superimposes digital information onto the user’s view of the physical environment. AR devices include head-mounted displays, mobile devices, tablets, and fixed projection systems. Within an HCDT, AR can present assembly instructions, maintenance guidance, robot trajectories, safety zones, quality information, predicted hazards, and system-status information directly within the worker’s field of view.

Rabah et al. (2018) combined digital-twin and AR technologies to support manufacturing visualization and interaction. Zhu et al. (2019) similarly used AR to visualize digital-twin data within manufacturing operations. These approaches reduce the need for workers to repeatedly shift attention between the physical task and a separate display.

AR can also support human-in-the-loop collaborative manufacturing. Li et al. (2022) developed an AR-assisted, digital-twin-enabled robot-collaboration system in which human input remained part of the operational loop. The interface allowed digital information about the robot and production system to be presented in the shared workplace, supporting coordination between the human and robot.

For safety applications, AR may display robot motion intentions, predicted paths, protective regions, approach warnings, or recommended escape directions. However, AR information should not obscure the physical robot, tool, product, or hazard. Safety-critical warnings must remain visible under changing illumination, worker movement, and partial tracking failure.

#### 5.5.3 Virtual Reality

Virtual Reality places the user inside a computer-generated environment and isolates the visual experience from the physical workplace. It can be used for production-system design, workstation evaluation, robot programming, operator training, maintenance rehearsal, and safety assessment without exposing workers or equipment to physical risk.

Havard et al. (2019) developed a digital-twin and VR co-simulation environment for designing and assessing industrial workstations. The virtual environment allowed users to examine production arrangements and human activities before physical implementation. Maragkos et al. (2019) used VR to support robot programming for human collaboration, demonstrating how an operator could develop or evaluate robot operations through an immersive interface.

VR has also been used to prototype proactive and adaptive human–robot collaboration. Matsas et al. (2018) developed VR-based methods for investigating how collaborative systems could respond to human behaviour in manufacturing. Malik et al. (2019) used immersive and collaborative virtual environments to support the design of human–robot workspaces.

Safety training represents another important application. Hazardous situations, emergency procedures, and unusual equipment failures can be reproduced without creating an actual threat. You et al. (2018) used immersive virtual environments to examine perceived safety during human–robot collaborative construction. VR training can also be connected to an HCDT so that task difficulty, instructional content, and feedback are adapted according to worker performance.

Nevertheless, successful performance in VR does not necessarily guarantee equivalent performance in the physical workplace. Differences in depth perception, field of view, haptic feedback, physical effort, motion latency, and risk perception may affect the transfer of training. Virtual training should therefore complement rather than completely replace supervised physical training.

#### 5.5.4 Mixed reality

Mixed Reality combines physical and virtual environments and allows digital and physical objects to interact. Unlike a basic AR overlay, an MR system attempts to maintain spatial relationships and interaction between the worker, physical equipment, and virtual content.

Su et al. (2022) developed an MR-enhanced human–robot interaction system using imitation-based mapping for intuitive tele-operation of a robotic arm and hand. The worker’s actions were mapped to robot commands while visual feedback supported control of the remote system. MR can similarly allow workers to manipulate virtual controls, adjust robot targets, inspect simulated trajectories, or interact with a virtual representation of equipment while observing the physical system.

MR interfaces can improve accessibility by allowing complex robot operations to be specified through natural spatial actions rather than conventional programming. However, registration errors between the physical and virtual environments can create serious risks. A virtually displayed robot position or safety boundary may not correspond exactly to its physical location. Safety-critical decisions should therefore not rely exclusively on non-safety-rated MR tracking.

#### 5.5.5 Gesture, Gaze, voice, and haptic interaction

Natural interaction methods can reduce dependence on keyboards, control panels, and handheld devices. Gesture recognition allows workers to issue commands through hand, arm, or body movement. Park et al. (2021) developed hands-free human-robot interaction using multi-modal gestures, deep learning, and wearable mixed reality. Such interfaces are useful when workers must keep their hands available for assembly or handling tasks. In recent years, deep-learning models have been shown to successfully detect hand gestures in real-time, including alphabetic and transactional gestures, and give visual explanations of their predictions Elgohr et al. (2025b). This work was carried out with the aim of sign-language recognition but it represents a potential solution for HCDT applications in which gesture recognition would be used for intuitive and accessible interfaces.

Gaze tracking may indicate the object, component, or robot to which the worker is attending. It can also support selection within AR or VR interfaces. However, gaze direction does not always represent intention, and workers may look at an object for inspection rather than command selection. Gaze-based control should therefore use confirmation mechanisms for consequential actions.

Voice interaction allows workers to request information, report problems, control equipment, or ask the HCDT to explain a recommendation. Conversational systems must manage turn-taking, interruption, confirmation, and recovery from misunderstood commands (Skantze, 2021). Industrial environments introduce additional difficulties because of machinery noise, hearing protection, multiple speakers, accents, and safety-critical terminology.

Haptic interfaces provide force, vibration, or tactile feedback. They can communicate contact, resistance, tool interaction, robot state, or proximity warnings when visual and auditory channels are occupied. Haptic feedback may be particularly useful for tele-operation and training, but its signals must be distinguishable and should not interfere with the physical task.

#### 5.5.6 Natural-language and generative-AI interfaces

Natural-language interfaces can make complex HCDT data accessible to workers who do not have specialist modelling or programming knowledge. An operator could ask the system why a robot stopped, request the predicted consequence of reallocating a task, retrieve a maintenance procedure, or ask which measurements indicate fatigue or ergonomic risk.

Language-model-based reasoning has been investigated for connecting high-level human instructions with robotic planning and environmental feedback. Huang et al. (2022) demonstrated an embodied reasoning approach in which language-based planning was updated using feedback from the surrounding environment. This direction suggests that future HCDTs may support conversational interaction with workers and supervisors.

However, generative-AI systems can produce incorrect, incomplete, or unsupported responses. A fluent answer should not be treated as evidence of technical correctness. In industrial applications, generated responses should be grounded in verified HCDT data, approved procedures, and current equipment states. Safety-critical commands should require explicit confirmation and be checked against operational and safety constraints before execution.

#### 5.5.7 Interface design and human factors

The effectiveness of an interface depends on the information presented, its timing, and the worker’s physical and cognitive context. Poor interface design may increase mental workload, obscure hazards, generate alarm fatigue, or encourage inappropriate reliance on automation. Masood and Egger (2019) identified cost, system complexity, device limitations, data security, privacy, and user acceptance as important barriers to industrial AR implementation.

The interface should prioritize information according to urgency, role, task, and consequence. Immediate collision warnings should be presented differently from long-term ergonomic recommendations or production-performance information. The number of simultaneous visual elements should be limited, and warnings should use consistent symbols, colours, sounds, and interaction patterns.

Personalization may adjust information density, terminology, language, font size, training support, or interaction method according to worker experience and accessibility requirements. However, personalization should not create inconsistent safety information between workers. Safety-critical indicators and emergency controls should remain standardized and immediately recognizable.

#### 5.5.8 Bidirectional human participation

A genuinely human-centric interface must support bidirectional communication. Workers should not be treated only as recipients of instructions generated by the HCDT. They should be able to correct an inaccurate posture or fatigue estimate, report discomfort, identify an unobserved hazard, reject an unsuitable task allocation, and request human assistance.

Worker feedback should be recorded together with the HCDT state and the reasoning behind the original recommendation. This information can support subsequent model evaluation and improvement. However, a single correction should not automatically retrain or modify a safety-critical model. Changes to operational models should pass through controlled validation and approval procedures.

The interface should also communicate which system currently holds decision authority. The worker must know whether a displayed action is advisory, awaiting confirmation, or already being implemented automatically. Clear representation of automation mode, decision responsibility, and available intervention mechanisms is necessary to prevent human-automation confusion.

### 5.6 Connectivity, edge-cloud computing, and interoperability

A HCDT requires continuous and reliable communication among workers, wearable sensors, environmental sensors, robots, machines, simulation models, and industrial information systems. Connectivity provides the data-exchange infrastructure through which the physical workplace updates its digital representation and the HCDT communicates recommendations or control actions back to the physical system.

The relevant technologies include the Industrial Internet of Things (IIoT), wired and wireless industrial networks, edge and fog computing, cloud platforms, service-oriented architectures, and standardized data interfaces. Their integration must satisfy different requirements for latency, bandwidth, reliability, security, interoperability, and computational capacity (Maddikunta et al., 2022; Qi et al., 2021).

#### 5.6.1 Industrial internet of things

The IIoT connects sensors, robots, machines, tools, products, and computing platforms within an industrial environment. In an HCDT, IIoT devices acquire and exchange information describing worker location, posture, physiological condition, environmental exposure, robot state, machine performance, and task progress Sbaragli et al. (2026).

The IIoT supports the bidirectional physical–digital connection required for a functional digital twin. Data from the physical environment update the virtual representation, while model outputs and operational decisions are transmitted back to interfaces, robots, and production-control systems. If communication is only unidirectional, the resulting system is more accurately described as a digital shadow than a complete operational digital twin.

Industrial connectivity can be implemented through Industrial Ethernet, Time-Sensitive Networking (TSN), 5G, Wi-Fi, Bluetooth Low Energy, Ultra-Wideband (UWB), Radio-Frequency Identification (RFID), and wireless body-area networks Khan et al. (2022). Different technologies may be used simultaneously because wearable sensing, robot control, worker localization, and enterprise-level analysis have different communication requirements Li and Li (2025); Urbaniak et al. (2024).

For example, Bluetooth Low Energy may be sufficient for periodically transmitting a worker’s heart rate, whereas robot collision avoidance requires substantially lower latency and greater reliability. Communication technology should therefore be selected according to the maximum permissible delay, required update frequency, data volume, energy consumption, and consequence of communication failure Urbaniak et al. (2024).

#### 5.6.2 Edge and fog computing

Edge computing performs data processing close to the worker, robot, sensor, or production cell. Rather than transmitting every raw measurement to a remote cloud platform, an edge device can filter signals, extract features, run AI models, detect unsafe conditions, and initiate time-sensitive responses locally.

Edge processing is particularly important for human-robot safety. Functions such as worker detection, proximity monitoring, collision prediction, and robot-speed adaptation may require responses within milliseconds. Depending entirely on a remote cloud service would introduce network delay and expose the safety function to internet or cloud-service interruptions.

Processing human data at the edge can also reduce privacy exposure. Raw images, physiological signals, or voice recordings can be analyzed locally, while only the resulting worker-state estimate is transmitted to the wider HCDT. For example, an edge device could convert a raw camera stream into skeletal coordinates or a posture-risk score without storing or transmitting identifiable video.

Fog computing provides an intermediate layer between local edge devices and centralized cloud infrastructure. A fog or plant-level server may coordinate information from several production cells, integrate multiple local twins, maintain plant-level models, and provide services that require more computing capacity than an individual edge device but lower latency than a remote cloud platform.

Edge and fog devices nevertheless have limited memory, processing power, and energy capacity. Lightweight AI models, efficient communication, hardware acceleration, and model-compression techniques may therefore be necessary. Their software and models must also be updated securely without interrupting safety-critical production.

#### 5.6.3 Cloud computing

Cloud computing provides scalable processing, storage, and access to computationally intensive services. Within an HCDT architecture, cloud resources can support long-term data storage, high-fidelity simulation, machine-learning model training, cross-site analysis, fleet-level digital twins, and comparison among production systems.

Cloud platforms can integrate HCDTs with Manufacturing Execution Systems (MES), Enterprise Resource Planning (ERP), maintenance-management systems, and supply-chain platforms. This integration allows human-related information to be considered together with production schedules, resource availability, maintenance conditions, and organizational objectives.

However, the cloud should not be treated as the only computing layer. Communication delays, bandwidth limitations, service outages, and cybersecurity events may make a cloud-only architecture unsuitable for real-time collaborative operations. Safety-critical control should remain local, while the cloud supports functions that can tolerate longer delays.

The division of responsibilities among edge, fog, and cloud platforms should be established according to the temporal and safety requirements of each HCDT function. Based on the timing, computational, and safety requirements reported in previous implementations (Kondo et al., 2024; Mungari et al., 2022; Urbaniak et al., 2024), a possible allocation is:

- Edge layer: sensor processing, worker localization, immediate hazard detection, collision avoidance, and local robot adaptation;
- Fog or plant layer: production-cell coordination, data fusion, plant-level simulation, and integration of multiple local twins; and
- Cloud layer: long-term storage, high-fidelity modelling, AI training, cross-site analysis, and organizational decision support.

#### 5.6.4 Real-time communication and synchronization

An HCDT must maintain an acceptably synchronized representation of the physical system. However, different variables change at different rates. Robot position may require millisecond-level updates, physiological measurements may be updated several times per second, and long-term ergonomic exposure may be evaluated over hours or days (Urbaniak et al., 2024).

The architecture should therefore define an update frequency and maximum acceptable data age for each variable. A timestamp must accompany every measurement, estimate, and model output. Without time synchronization, the HCDT may combine a current robot position with an outdated worker location and consequently produce an unsafe recommendation.

Laaki et al. (2019) demonstrated the development of a digital twin for real-time remote control over mobile communication networks. The study highlighted the importance of network performance, communication architecture, and synchronization when a digital representation is used to influence a physical system remotely.

Network jitter, packet loss, clock drift, and out-of-order messages must also be considered. Safety-critical components should detect stale or missing information and transition to a predefined safe state. An HCDT should not continue operating as if synchronization were valid when communication quality falls below an acceptable threshold.

#### 5.6.5 Co-simulation and distributed digital twins

HCDTs may include several distributed models representing workers, robots, machines, manufacturing cells, production flow, and organizational processes. These models may execute on different hardware and software platforms. Distributed communication is therefore required to coordinate model inputs, outputs, simulation time, and state changes.

Leng et al. (2019) developed a digital-twin-driven manufacturing cyber–physical system for the parallel control of a smart workshop. The framework demonstrated how physical and virtual production systems can be connected for monitoring and operational control. Ding et al. (2019) similarly defined a digital-twin-based cyber–physical production system for autonomous manufacturing.

Müller et al. (2021) combined material-flow simulation, digital twins of manufacturing cells, an Automated Guided Vehicle (AGV), and a mixed-reality application in real time. This demonstrates the need to exchange information across simulation, control, visualization, and physical-system components.

In an HCDT, distributed models may operate at different temporal resolutions. A robot-control model may be updated every few milliseconds, while a production-flow model may update after the completion of each task. Synchronization mechanisms must prevent a slow model from blocking a time-critical safety function.

#### 5.6.6 Interoperability

Interoperability is the ability of different devices, software platforms, and organizational systems to exchange and correctly interpret information. It is a major challenge because HCDTs combine data from wearable devices, cameras, robots, simulation tools, control systems, databases, and enterprise applications.

Technical interoperability ensures that systems can exchange data through compatible protocols and interfaces. Syntactic interoperability establishes a common data format, while semantic interoperability ensures that the exchanged information has the same meaning to all participating systems.

For example, two systems may both exchange a variable called “worker load,” but one may represent the mass being carried while the other represents estimated cognitive workload. Even though the data formats are technically compatible, the meaning is inconsistent. Semantic descriptions, metadata, ontologies, units, coordinate systems, and standardized naming are therefore necessary.

Qi et al. (2021) identified modelling, simulation, communication, data fusion, interaction, and service technologies as interconnected enablers of DTs. Perno et al. (2022) found that system integration, real-time data exchange, data management, intellectual-property protection, and organizational barriers remain important challenges in industrial DT implementation.

Interoperability frameworks should define:

- common identifiers for workers, robots, machines, tasks, and products;
- coordinate systems and unit conventions;
- timestamps, sampling rates, and synchronization rules;
- data-quality and uncertainty descriptors;
- model and software-version information;
- access rights and privacy classifications; and
- interfaces for reading, updating, and controlling twin components.

#### 5.6.7 Reliability and degraded operation

A human-centric architecture must remain safe when connectivity or computing resources fail. Loss of a wearable sensor, edge device, network connection, cloud service, or simulation model should not produce uncontrolled robot behaviour.

Each HCDT function should therefore define a degraded operating mode. If a non-critical ergonomic sensor becomes unavailable, the system may continue operating while notifying the worker. If worker-localization data are lost during close HRC, the robot may need to reduce speed or stop. If worker-localization data are lost during close HRC, the robot may need to reduce speed or stop. The response should reflect the consequence of operating without the missing information (Li and Li, 2025; Urbaniak et al., 2024).

Local components should retain the minimum models and safety rules required for independent safe operation. Cloud disconnection may temporarily remove optimization or long-term predictive services, but it should not disable emergency stopping, separation monitoring, or basic production control. This separation between safety-critical local functions and higher-level cloud services improves both resilience and worker protection (Kondo et al., 2024; Urbaniak et al., 2024).

### 5.7 Integration of the enabling technologies

The technologies discussed in the preceding subsections should not be treated as independent components. A functional HCDT emerges from their integration into a continuous human-cyber-physical loop. This loop connects the worker, collaborative robot, production equipment, industrial environment, digital models, and decision-support mechanisms. Its purpose is to ensure that human and operational conditions influence manufacturing decisions and that the consequences of those decisions are continuously evaluated (Asad et al., 2023a; Qi et al., 2021; Baratta et al., 2024).

Figure 5 presents the proposed six-stage HCDT framework, derived through qualitative synthesis of recurring requirements in the reviewed literature. Its novelty lies in integrating technical functions with worker benefits, human authority, governance, uncertainty, and safe operation. The six stages are: (1) sensing and data acquisition, (2) communication and synchronization, (3) digital modelling and state representation, (4) AI-based analysis and prediction, (5) decision-making and physical adaptation, and (6) human and system feedback. Together, these stages form a continuous human-cyber-physical loop protected by cybersecurity, privacy, and secure data-governance mechanisms.

**FIGURE 5:**
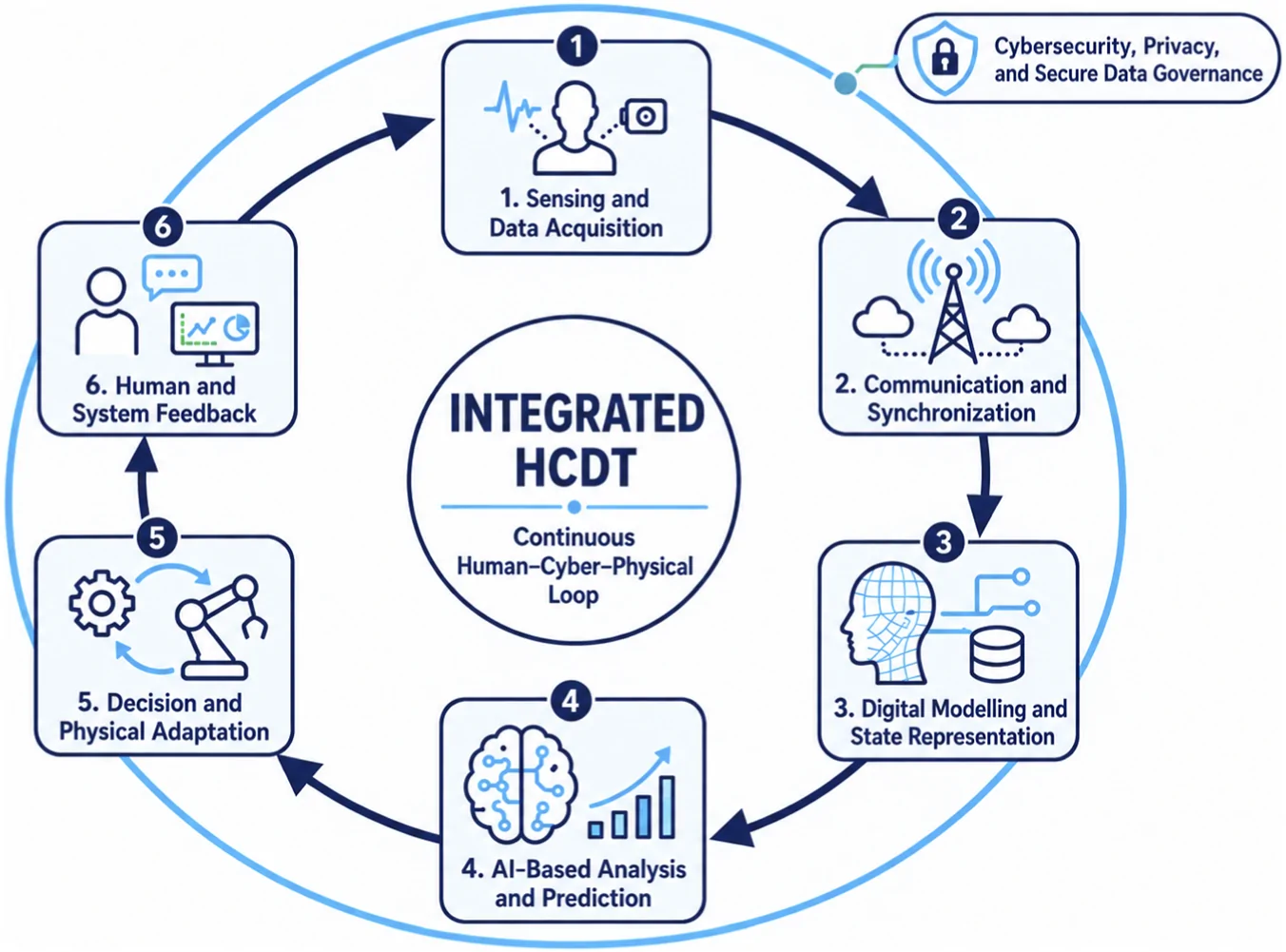
Proposed literature-derived conceptual framework for closed-loop HCDT operation. Multi-modal sensing is intended to update a synchronized digital representation supporting AI-based analysis, decision-making, physical adaptation, and continuous human and system feedback. The complete architecture requires future validation.

To operationalize the three I5.0 pillars, the proposed framework incorporates a multi-objective evaluation process within the transition from AI-based analysis to physical adaptation. Before an action is implemented, alternative decisions should be evaluated jointly in terms of worker safety, productivity, computational and energy cost, environmental impact, and system resilience. These criteria may be formulated as objectives or constraints according to the application. Because improvements in one criterion may negatively affect another, the HCDT should explicitly present the resulting trade-offs rather than combine them into a single performance measure. The outcome of this evaluation informs the selection of an appropriate action before its implementation in the physical industrial environment.

#### 5.7.1 Cybersecurity, privacy, and trustworthy HCDT operation

Although cybersecurity is not an individual enabling technology, it is a cross-cutting architectural capability required for the trustworthy integration of all HCDT technologies. An operational HCDT may connect wearable and physiological sensors, cameras, collaborative robots, industrial controllers, edge devices, cloud platforms, AI models, simulations, and Human-Machine Interfaces. Because these components exchange data and control commands, a cybersecurity failure could affect worker privacy, operational continuity, human autonomy, and physical safety.

Cybersecurity consequently supports I5.0 resilience by enabling an industrial system to anticipate, withstand, respond to, and recover from disruptions. For an HCDT, resilience includes maintaining safe operation when devices, data, models, or communication channels become compromised.

##### 5.7.1.1 Cybersecurity threats affecting HCDTs

The HCDT attack surface extends across its sensing, communication, computation, modelling, analytical, and actuation layers. Attackers may compromise wearable devices, manipulate camera streams, inject false physiological measurements, replay recorded data, intercept communications, modify control commands, or disrupt connectivity through denial-of-service attacks. Edge and cloud platforms may also be affected by unauthorized access, malware, compromised software updates, and incorrectly configured interfaces.

The Digital Twin itself may become an attack target. An adversary could modify worker profiles, operational constraints, model parameters, simulation results, or AI confidence estimates. Training-data poisoning, adversarial inputs, model extraction, and replacement of a validated model with an unauthorized version may also affect system decisions. At the physical layer, manipulated commands could change robot speed, trajectory, force, task allocation, or stopping behaviour.

Khalid et al. (2018) developed a security framework for collaborative robotic cyber-physical systems that combines authentication and data-integrity verification with an independent mechanism for comparing real-time sensor measurements against stored system specifications. This is applicable to HCDTs because authentication alone does not establish that received information is physically plausible or operationally safe.

For example, a compromised but authenticated sensor may report an incorrect worker position, while a modified model may classify an unsafe robot trajectory as acceptable. HCDTs should therefore combine conventional cybersecurity controls with contextual validation based on physical constraints, temporal consistency, historical behaviour, redundant measurements, and expected system states.

##### 5.7.1.2 Human-centered cybersecurity and privacy

Cybersecurity is particularly important in HCDTs because the processed information may describe identifiable workers. The system may collect physiological signals, body movements, location histories, facial images, voice recordings, workload estimates, fatigue levels, ergonomic risks, and performance-related information. Unauthorized disclosure or secondary use could lead to surveillance, discrimination, inappropriate employee evaluation, or loss of autonomy.

Cybersecurity and privacy are related but distinct. Cybersecurity protects information against unauthorized access, manipulation, disruption, and destruction. Privacy determines whether personal information should be collected, how it may be used, who may access it, and how long it should be retained. An HCDT may be technically secure but still violate privacy by collecting excessive data or using them beyond their accepted purpose. Zhang and Tai connected human-centered Digital Twin interaction with resilience against cyber vulnerabilities in a medical application (Zhang and Tai, 2022). Although the domain is healthcare, its principles are transferable to industrial HCDTs that process physiological and behavioral worker information.

A human-centered security strategy should include informed consent, data minimization, purpose limitation, privacy-preserving processing, access auditing, and defined retention periods. Workers should know which measurements are collected, which characteristics are inferred, who can access the information, and whether it may influence safety interventions, task allocation, productivity assessment, or employment-related decisions.

##### 5.7.1.3 Defence-in-depth architecture

No single security mechanism can protect the complete HCDT loop. Protection should therefore be distributed across the architecture:

- Device security: Sensors, wearables, robots, controllers, and edge platforms should have verifiable identities. Secure boot, signed firmware, protected credentials, device authentication, and controlled updates can reduce unauthorized access.
- Communication security: Data should be encrypted during transmission, while message authentication and integrity verification should detect modification. Timestamps, sequence numbers, or cryptographic nonces can protect against replay attacks.
- Access control: Access should follow authenticated identities and minimum-privilege principles. Maintenance engineers, safety supervisors, researchers, and production managers should not receive identical access to worker data, models, and control commands.
- Network segmentation: Wearable networks, robot-control systems, enterprise platforms, and cloud services should not form an unrestricted communication environment. Segmentation can contain compromised devices and isolate safety-critical components.
- Data and model integrity: Sensor measurements, worker profiles, AI parameters, Digital Twin models, and safety constraints should be protected against unauthorized modification. Digital signatures, cryptographic hashes, controlled repositories, and model-version records can establish provenance and support restoration.
- Anomaly detection: Network traffic, authentication records, sensor measurements, model outputs, and physical responses should be analyzed together. The Digital Twin can help determine whether observed behaviour is consistent with expected physical and operational conditions.
- Incident response: The architecture should define how attacks are detected, contained, investigated, and recovered from. Security logs should provide traceability between affected data, decisions, models, and physical actions.

Erceylan et al. (2025) showed that DTs can support continuous threat modelling through system analysis, simulation, and state replication across the cyber–physical-system lifecycle. This is relevant because the HCDT attack surface changes whenever sensors, models, workers, software versions, and communication pathways are updated.

DT simulations can also evaluate attack scenarios without exposing workers or equipment to unnecessary risk. Dietz et al. (2020) proposed integrating DT security simulations with Security Operations Centre processes. Recent reviews similarly identify attack simulation, anomaly detection, predictive risk assessment, and incident response as security-enhancing DT applications (Homaei et al., 2024; Kampourakis et al., 2025).

However, a Digital Twin should not automatically be considered trustworthy because it is used for security. Its data, models, synchronization mechanisms, and simulation environments may themselves be compromised. Security-enhancing DTs should supplement established industrial cybersecurity controls and certified safety functions rather than replace them.

##### 5.7.1.4 Blockchain and secure data exchange

Blockchain has been proposed to improve the integrity, provenance, and traceability of Digital Twin information. Suhail et al. demonstrated how blockchain-based DTs could enhance situational awareness and data reliability in cyber–physical systems (Suhail et al., 2022). It could provide tamper-evident records of model updates, access events, software versions, and decisions generated by distributed HCDT components.

Alcaraz et al. proposed Digital Twin communities for controlled sharing of simulation results across organizations (Alcaraz et al., 2025). This may be relevant when HCDT models or aggregated safety information are shared among manufacturers, technology providers, research institutions, and production sites.

Nevertheless, blockchain is not a complete cybersecurity solution. It may protect integrity and traceability, but it cannot verify that an original sensor measurement is correct or prevent a compromised device from submitting false information. It may also introduce latency, computational overhead, and data-erasure difficulties. Sensitive physiological or biometric information should therefore not be stored directly on an immutable ledger. More appropriate applications include recording hashes, permissions, access events, model versions, and non-identifiable audit information.

##### 5.7.1.5 Cybersecurity-aware degraded operation

Cybersecurity incidents should be incorporated into the HCDT degraded-operation strategy. If the authenticity, integrity, or freshness of safety-critical information cannot be established, the system should not continue operating as though the information were trustworthy. It should enter a predefined safe state according to the consequence of the affected function.

Loss of trust in a non-critical ergonomic record may require notification and temporary suspension of the analysis. In contrast, uncertainty about worker-location data during close human-robot collaboration may require reduced robot speed, increased separation distance, rejection of remote commands, transfer of authority to a local safety controller, or a controlled stop.

The HCDT should distinguish, where possible, among sensor faults, communication failures, model errors, and malicious manipulation, although their immediate safety responses may be similar. It should preserve sufficient evidence for investigation without unnecessarily exposing worker information. Workers and supervisors should also receive understandable information about the affected function, the reason for the protective action, and the person or system currently holding decision authority.

Cybersecurity supports human-centricity by protecting worker information, physical safety, autonomy, and authority; resilience through detection, containment, recovery, and safe degraded operation; and sustainability by reducing cyber-related equipment damage, production waste, and resource-intensive recovery. A trustworthy HCDT must therefore be secure by design, privacy-aware, continuously monitored, and capable of protecting workers when trusted operation cannot be maintained.

#### 5.7.2 Sensing and data acquisition

The integration process begins with the acquisition of data from the worker, robot, machine, task, and industrial environment. Human-focused sensors provide information about posture, movement, location, physiological condition, workload, and interaction behaviour. Robot and machine sensors provide information about position, velocity, force, process state, equipment condition, and task progress. Environmental sensors measure factors such as temperature, humidity, illumination, noise, vibration, dust, and hazardous gases.

These measurements should be time-stamped and accompanied by information describing their quality, sampling frequency, and uncertainty. A measured value should not automatically be considered a complete representation of the worker. For example, an increased heart rate may result from physical effort, psychological stress, thermal exposure, or normal movement. Human, task, and environmental information must therefore be combined before the HCDT assigns meaning to the measurement.

#### 5.7.3 Communication and synchronization

The acquired data are transmitted through industrial communication networks to edge, fog, or cloud computing resources. The communication layer connects physical devices with the digital representation and maintains synchronization between them. Different data streams require different update frequencies. Robot location and worker proximity may require millisecond-level updates, whereas ergonomic exposure and long-term fatigue can be evaluated over longer periods.

Edge computing supports immediate sensor processing and time-critical responses. Plant-level or fog platforms can integrate information from multiple workstations, while cloud computing supports long-term storage, computationally intensive simulation, and AI-model training. The allocation of functions across these layers should reflect their latency, computational, safety, and privacy requirements (Maddikunta et al., 2022; Laaki et al., 2019).

Communication failure must not result in uncontrolled behaviour. If a critical data stream becomes unavailable or outdated, the HCDT should identify the loss of synchronization and move the affected operation towards a predefined safe state.

#### 5.7.4 Digital modelling and state representation

The modelling layer converts synchronized data into virtual representations of the worker, robot, task, equipment, and environment. These representations may include geometric models, biomechanical models, robot models, process simulations, and data-driven models. Their integration creates a contextual description of the complete human-cyber-physical system rather than an isolated model of the worker or machine.

The human component may represent posture, physical capability, fatigue, workload, experience, and interaction behaviour. The robot component represents its configuration, trajectory, operational mode, payload, and safety state. The production component includes the task sequence, equipment condition, material flow, and production objectives.

The required model fidelity depends on the decision being supported. Detailed biomechanical or finite-element models may be appropriate for offline safety assessment, whereas reduced-order or surrogate models are required for real-time adaptation. Multi-fidelity modelling allows the HCDT to combine detailed offline analysis with computationally efficient online operation (Kapteyn, 2021; Calka et al., 2021).

#### 5.7.5 AI-based analysis and prediction

AI and computational-intelligence methods analyze the integrated HCDT state. Their functions may include human-activity recognition, posture assessment, intention prediction, fatigue detection, anomaly detection, collision prediction, equipment-failure prediction, and estimation of future production conditions.

The analytical layer should distinguish between measured, estimated, and predicted information. A worker’s current location may be directly measured, while fatigue or intention is inferred from several uncertain signals. The resulting decision should reflect the confidence associated with each estimate.

The HCDT can also evaluate alternative actions before implementation. For example, it may simulate the effects of reducing robot speed, changing its trajectory, reallocating a task, modifying the workstation, or providing additional instructions. Each alternative can be evaluated against productivity, worker safety, ergonomic loading, energy consumption, and production resilience.

Human-centered objectives should be included explicitly. An AI model trained only to minimize cycle time may increase worker workload or reduce recovery time. Worker safety, wellbeing, autonomy, and capability should therefore be represented as decision criteria rather than assumed to emerge automatically from production optimization.

#### 5.7.6 Decision and physical adaptation

After analyzing the current and predicted states, the HCDT can provide a recommendation or initiate an adaptation. Potential actions include:

- modifying robot speed or trajectory;
- increasing the separation distance between the worker and robot;
- changing the robot’s assistance level;
- reallocating tasks between the worker and robot;
- presenting maintenance, assembly, or safety instructions;
- modifying the production schedule;
- recommending a rest period or ergonomic intervention; and
- stopping an operation when an unacceptable risk is detected.

The authority assigned to the HCDT should depend on the consequence and reversibility of the action. Low-risk and reversible adjustments may be implemented automatically within validated limits. Actions affecting worker safety, health, employment evaluation, or major production decisions should retain appropriate human oversight.

Uncertainty should be carried through the HCDT process, and should not be judged on a stage-by-stage basis. Data quality and reliability descriptors should be captured by sensors and their weight in multi-modal fusion should reflect this. This fused representation should then be used to estimate human states and future conditions with calibrated confidence and/or probability values. These uncertainty estimates should then be included with the inputs and outputs of simulation, prediction and decision making elements. Uncertainty measures obtained from different models/sensors are not necessarily comparable; they need to be calibrated and combined based on the supported decision and decision context. For the adaptation authority, the resulting decision confidence and the proposed action severity and reversibility should determine whether: high-confidence, low-risk action is taken automatically, within validated limits; intermediate-confidence action is taken under supervision or human confirmation; or low-confidence, safety-related outputs trigger conservative or degraded operation.

Collaborative robots and production-control systems implement physical adaptations, while AR, VR, dashboards, voice interfaces, and other HMIs communicate recommendations to workers. The interface should clearly indicate whether an action is advisory, awaiting confirmation, or already being implemented (Li et al., 2022).

#### 5.7.7 Human and system feedback

After an adaptation is implemented, the sensing layer measures its effect on the worker and physical system. This closes the HCDT loop. The system can determine whether the action reduced risk, improved posture, maintained production performance, or created an unintended consequence.

Worker feedback is an essential part of this process. The worker should be able to report discomfort, reject an unsuitable recommendation, identify an unobserved hazard, and correct an inaccurate HCDT representation. This input may contain contextual knowledge that cannot be obtained through sensors.

The outcome of each action should be recorded together with the HCDT state, the selected decision, and the worker response. These records support model evaluation and subsequent improvement. However, safety-critical models should not be modified automatically from a single event. Model changes should pass through controlled verification and validation before deployment.

#### 5.7.8 Integrated closed-loop operation

A complete HCDT closed loop distinguishes an operational HCDT from a static human model or a unidirectional monitoring system. A static model may describe a worker, while a monitoring system may transfer data from the worker to a digital platform. An HCDT additionally maintains synchronization, interprets the worker within the industrial context, evaluates alternative actions, and supports feedback to the physical system. Nevertheless, the presence of a closed data loop does not by itself establish human-centricity. A system may continuously monitor a worker and adapt production exclusively to increase output. For the integrated architecture to be genuinely human-centric, human information must influence decisions that produce demonstrable benefits in safety, ergonomics, wellbeing, accessibility, autonomy, or skill development.

#### 5.7.9 Requirements for practical integration

Practical implementation requires the integrated system to satisfy the following requirements:

- Personalization: the human model should represent the relevant characteristics of an individual worker rather than only a generic population model;
- Continuous updating: the HCDT state should be updated at a frequency appropriate to the supported decision;
- Context awareness: human information should be interpreted together with task, robot, machine, and environmental conditions;
- Interoperability: heterogeneous devices and models should exchange information using consistent formats, units, identifiers, and semantic definitions;
- Uncertainty awareness: measurements, predictions, and simulation results should include confidence and validity information;
- Fail-safe operation: communication, sensing, or model failure should lead to a defined safe or degraded operating mode;
- Human authority: workers should retain an appropriate ability to understand, correct, reject, or override system decisions; and
- Demonstrable worker benefit: human data should support safety, wellbeing, autonomy, accessibility, or capability rather than worker monitoring alone.

Together, these requirements define the intended integration of human, robot, DT, AI, and manufacturing components within the proposed conceptual framework. The framework describes how data acquisition, interpretation, decision-making, adaptation, and feedback could be coordinated in a closed loop. Whether this organization can be implemented effectively and deliver measurable worker benefits remains to be evaluated.

## 6 Discussion

The findings are synthesized below in relation to the four research questions introduced in Section 1.

### 6.1 RQ1: distinguishing HCDTs from related concepts

The synthesis of the reviewed literature indicates that HCDTs should not be regarded as conventional Digital Twins that merely incorporate worker data. Instead, an HCDT uses human and operational information to support decisions that provide explicit and measurable benefits to workers, including improvements in safety, ergonomics, wellbeing, autonomy, accessibility, and skill development (Krupáš et al., 2024). Consequently, the inclusion of wearable sensors, a human model, or a collaborative robot does not by itself make an industrial system human-centric. A system that continuously monitors workers primarily to increase productivity may contain human-related data, but it does not satisfy the worker-centered objectives of an HCDT (Fontes et al., 2024; Ball, 2010).

The reviewed literature also clarifies the relationships among human models, Human Digital Twins (HDTs), Human-Robot Collaboration (HRC), and HCDTs. A conventional human model may represent a specific characteristic, such as posture, motion, anthropometry, or physical capacity. An HDT provides a more individualized and dynamically updated digital representation of a worker, whereas HRC primarily concerns the physical or cognitive interaction between humans and robots. An HCDT extends these concepts by integrating information about the worker, robot, task, equipment, production process, and industrial environment within a continuous human-cyber-physical loop (He et al., 2024; Baratta et al., 2024; Nie et al., 2026). This integration enables data exchange, state estimation, prediction, simulation, decision-making, physical adaptation, and worker feedback. Based on this synthesis, we define an HCDT by how human information influences industrial decisions and worker outcomes, rather than solely by the technologies included in the system.

### 6.2 RQ2: integration of sensing, AI, industrial data, and simulation

The findings further indicate that no single enabling technology is sufficient to create an operational HCDT. Human-sensing technologies can capture movement, posture, location, physiological signals, and environmental exposure; however, these measurements can be ambiguous when interpreted without contextual information (Tiberio et al., 2013). For example, an increased heart rate may be associated with physical exertion, psychological stress, thermal exposure, or ordinary movement. Similarly, physiological signals such as heart-rate variability, electrodermal activity, electromyography, respiration, and skin temperature provide indirect information about human condition rather than unique measurements of a particular psychological or cognitive state (Hakonen et al., 2015). The evidence therefore supports combining human measurements with information about the task, machine, robot, and surrounding environment. Such integration requires multi-modal data fusion, temporal synchronization, contextual modelling, data-quality assessment, and interoperable representations within the HCDT.

The reviewed studies demonstrate that AI can support activity recognition, fatigue and workload estimation, intention prediction, collision detection, anomaly detection, and industrial decision support (Tiberio et al., 2013; Hakonen et al., 2015; Krupáš et al., 2024). Nevertheless, fatigue, stress, workload, and intention are generally latent human states inferred from physiological, behavioral, contextual, or multi-modal observations rather than directly measured quantities. The reliability of these estimates may vary across workers, tasks, sensing conditions, and industrial environments. Moreover, conventional AI models may produce poorly calibrated confidence values or fail to represent important sources of data and model uncertainty (Gawlikowski et al., 2023). Based on these limitations, we propose that HCDTs explicitly distinguish among directly measured variables, estimated human states, and predicted future conditions. We further recommend that predictions be accompanied by confidence or uncertainty information and that low-confidence, safety-related decisions require human confirmation or trigger a predefined conservative operating mode. These are design recommendations derived from the reviewed evidence and require validation in operational HCDT applications.

### 6.3 RQ3: trustworthy closed-loop adaptation

The reviewed literature also shows that HCDTs can support predictive and adaptive interventions, including changes to robot speed, trajectory, separation distance, task allocation, workstation configuration, safety information, and rest recommendations (Liao and Cai, 2024). Simulation and Digital Twin models can additionally be used to evaluate alternative interventions before they are introduced into the physical workplace (Cimino et al., 2024). However, the existence of these technical capabilities does not establish that every intervention should be executed autonomously. Research on human interaction with automation demonstrates that the appropriate level of automation depends on the function being automated and its consequences for human performance and authority (Parasuraman et al., 2000). Safe HRC similarly requires consideration of the complete collaborative system, including the robot, worker, task, workspace, tools, control system, and potential failure conditions (Lasota et al., 2017; Gualtieri et al., 2022).

Accordingly, we propose that the degree of HCDT automation should be proportional to the severity, uncertainty, and reversibility of the decision. Low-risk and readily reversible adaptations may be automated, whereas decisions that could substantially affect worker health, safety, employment, or autonomy should retain meaningful human oversight. Workers should also be able to understand, question, correct, refuse, or challenge consequential system recommendations. These principles represent normative recommendations of the proposed framework rather than capabilities that have already been demonstrated consistently across the reviewed HCDT implementations.

Ethical and organizational considerations are equally important for practical implementation. HCDTs may collect sensitive physiological, behavioral, location, biometric, and performance-related information (Fontes et al., 2024). Workplace-surveillance research demonstrates that employee-monitoring technologies can affect privacy, trust, autonomy, employment relationships, and organizational power when their purposes and governance are insufficiently defined (Ball, 2010). HCDTs could therefore be repurposed for continuous surveillance, secondary use of worker data, or unfair performance assessment even when the underlying system is technically secure. cybersecurity and privacy should consequently be treated as related but distinct requirements: cybersecurity protects information and system operation from unauthorized access or manipulation, whereas privacy concerns whether human data should be collected, processed, retained, or reused in the first place.

Based on these risks, we recommend purpose limitation, data minimization, informed worker participation, role-based access control, defined retention periods, secure processing, and explicit policies for data access, ownership, correction, and deletion. Workers should be informed about which variables are directly measured, which human states are inferred, how their data influence decisions, and who can access the resulting information. Privacy protection, explainability, cybersecurity, worker involvement, and mechanisms for contesting consequential decisions should therefore be incorporated during HCDT design rather than added retrospectively (Fontes et al., 2024). These governance measures are literature-informed recommendations whose effectiveness should be assessed with workers and other stakeholders in real industrial environments.

### 6.4 RQ4: requirements for measurable worker-centered benefits

Important gaps nevertheless remain in the available literature. The reviewed studies include conceptual frameworks, laboratory experiments, simulation studies, and small-scale prototypes, while evidence from longitudinal industrial deployment remains limited (Krupáš et al., 2024; Baratta et al., 2024). Existing evaluations frequently emphasize model accuracy or production performance without directly measuring sustained benefits for workers. Comparison across studies is also complicated by inconsistent terminology, system boundaries, datasets, and evaluation metrics. The reviewed evidence therefore supports the development of standardized reporting and validation procedures that jointly assess technical performance and worker-centered outcomes. Worker-centered outcomes should be evaluated using indicators appropriate to the application. Safety may be assessed through injury, near-miss, collision-risk, and hazardous-exposure rates; ergonomics through RULA, REBA, posture exposure, and muscular load (McAtamney and Corlett, 1993; Hignett and McAtamney, 2000); and workload through validated instruments such as NASA-TLX (Hart and Staveland, 1988). Autonomy may be evaluated through decision authority, override frequency, perceived control, and validated work-design measures (Morgeson and Humphrey, 2006), while accessibility and skill development may be assessed through task-completion disparities, usability, competency, error reduction, learning time, and skill retention. These indicators should be compared before and after HCDT intervention, or against an appropriate baseline, alongside technical and production measures.

A specific limitation of the proposed multilayer architecture is that it is derived from the reviewed literature and has not yet been empirically validated or systematically assessed against representative published HCDT implementations. Its contribution should therefore be understood as a conceptual synthesis of functional components, integration requirements, and human-centered design considerations rather than as a validated operational architecture. Future research should assess its coverage and applicability through structured mapping to published implementations, followed by prototype-based and industrial evaluation of the complete closed loop. Such evaluation is required before conclusions can be drawn regarding its operational effectiveness, generalizability, or readiness for deployment.

Future studies should evaluate complete closed-loop HCDTs under realistic industrial conditions. Technical validation should include accuracy, calibration, latency, reliability, robustness, interoperability, cybersecurity, and degraded-mode operation. Worker-centered evaluation should examine safety, ergonomic exposure, workload, wellbeing, trust, autonomy, accessibility, acceptance, and opportunities for skill development. Longitudinal studies are particularly important because interventions that produce short-term improvements may also introduce surveillance, cognitive burden, over-reliance on automation, loss of autonomy, or previously unrecognized safety risks (Ball, 2010; Parasuraman et al., 2000). Overall, the reviewed literature suggests that HCDTs could provide an important enabling architecture for I5.0. Their success, however, should be determined by whether they produce demonstrable and sustained benefits for workers, rather than solely by their technical sophistication or contribution to production performance.

## 7 Conclusion

Human-Centric Digital Twins (HCDT) represent an important enabling architecture for translating the principles of Industry 5.0 (I5.0) into operational industrial systems. This review has shown that an HCDT cannot be created through a single model, sensor, or AI method. It emerges from the coordinated integration of human-focused sensing, artificial and computational intelligence, digital modelling and simulation, collaborative robotics and adaptive control, visualization and extended-reality interfaces, and industrial connectivity, edge–cloud computing, and interoperability. Together, these technologies establish a continuous human–cyber–physical loop linking data acquisition, synchronization, state representation, analysis, decision-making, physical adaptation, and feedback.

The review also distinguishes HCDTs from conventional human models, Human Digital Twins (HDTs), and Human–Robot Collaboration systems. A digital representation of a worker does not become human-centric merely because it includes human data. Similarly, automated adaptation does not necessarily benefit the individual affected by it. A genuine HCDT must use human and operational information to support decisions that produce measurable improvements in safety, ergonomics, wellbeing, autonomy, accessibility, or skill development. Worker feedback and appropriate human authority must therefore remain integral to the closed-loop architecture. Privacy, transparency, explainability, uncertainty awareness, proportional data collection, interoperability, and safe degraded operation are also essential requirements for practical deployment.

The proposed literature-derived conceptual framework organizes HCDT components and integration requirements to inform future research, system design, and comparison. Its practical applicability, operational effectiveness, and ability to deliver measurable worker benefits remain to be validated. Future research should move beyond isolated prototypes and demonstrate complete closed-loop systems under realistic industrial conditions. Particular attention is required for multi-modal data fusion, personalized and adaptive human models, prediction under uncertainty, simulation-to-reality transfer, long-term model updating, and the validation of human-centered outcomes. Standardized evaluation methods and longitudinal industrial studies are needed to determine whether HCDTs deliver sustained benefits without introducing excessive surveillance, cognitive burden, loss of autonomy, or new safety risks. By connecting enabling technologies with explicit human outcomes, HCDTs can support the development of more adaptive, sustainable, resilient, and genuinely human-centric industrial systems.
